# A pathophysiological view of the long non-coding RNA world

**DOI:** 10.18632/oncotarget.2770

**Published:** 2014-11-15

**Authors:** Federico Di Gesualdo, Sergio Capaccioli, Matteo Lulli

**Affiliations:** ^1^ Department of Experimental and Clinical Biomedical Sciences, University of Florence, Italy

**Keywords:** cellular homeostasis, lncRNA dysregulation, cancer hallmarks

## Abstract

Because cells are constantly exposed to micro-environmental changes, they require the ability to adapt to maintain a dynamic equilibrium. Proteins are considered critical for the regulation of gene expression, which is a fundamental process in determining the cellular responses to stimuli. Recently, revolutionary findings in RNA research and the advent of high-throughput genomic technologies have revealed a pervasive transcription of the human genome, which generates many long non-coding RNAs (lncRNAs) whose roles are largely undefined. However, there is evidence that lncRNAs are involved in several cellular physiological processes such as adaptation to stresses, cell differentiation, maintenance of pluripotency and apoptosis. The correct balance of lncRNA levels is crucial for the maintenance of cellular equilibrium, and the dysregulation of lncRNA expression is linked to many disorders; certain transcripts are useful prognostic markers for some of these pathologies. This review revisits the classic concept of cellular homeostasis from the perspective of lncRNAs specifically to understand how this novel class of molecules contributes to cellular balance and how its dysregulated expression can lead to the onset of pathologies such as cancer.

## INTRODUCTION

Cellular homeostasis is a delicate condition that requires the ability of cells to adapt to minimal variation to maintain a “dynamic equilibrium”. Many factors such as nutrient availability and growth or death stimuli provoke diverse cellular responses; for example, cells can be induced to adjust their metabolic state, proliferate, differentiate or undergo apoptosis [1]. The regulation of gene expression is a crucial event in determining the cellular response to micro-environmental changes, and it is tightly controlled by specific factors, which are classically considered to be proteins. RNA, with the exception of tRNAs and rRNAs, has generally been considered an intermediate between DNA, the “sanctum sanctorum” of life, and proteins, the molecules through which life is expressed; therefore, the importance of RNA has been restricted to its coding role [2]. However, the discovery of RNA interference (RNAi) provided impetus to the identification and characterization of regulatory non-coding RNAs. Over the last two decades, the improvement in high-throughput technologies has led to the detection of many long non-coding RNAs (lncRNAs). Although the vast majority of their functions remain unexplored, there is evidence that some lncRNAs are involved in physiological processes that maintain cellular and tissue homeostasis, and that consequently, the dysregulated expression of lncRNAs contributes to the onset and progression of many pathological conditions. Furthermore, a recent study demonstrated that the genetic knockout of some lncRNAs in mice resulted in peri- or post-natal lethality or developmental defects [3], consistent with the idea that the sequences that were previously considered “junk DNA” are essential for life. This review aims to advance the classic physiopathological view of cellular homeostasis by focusing on lncRNAs, highlighting how their balanced expression is crucial for the maintenance of cellular equilibrium and how their dysregulation contributes to the onset and progression of human pathologies.

### Classification of lncRNAs: a brief update on an intricate scenario

**Figure 1:**
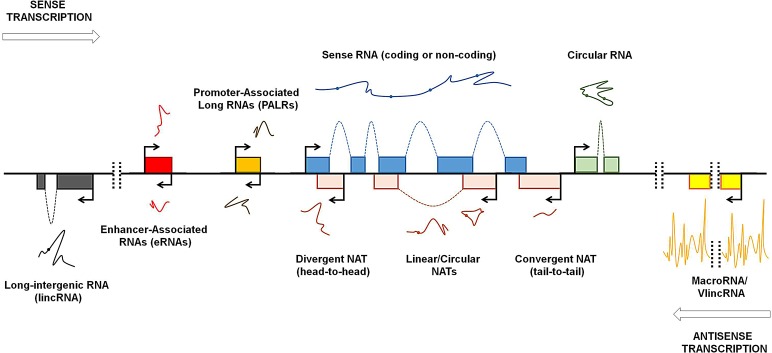
Genomic organization of lncRNAs Pervasive transcription of the genome occurs bidirectionally (arrows). Exons are schematically represented with colored boxes. Spliced transcripts are represented with lines; spots represent splice sites. LncRNAs that are transcribed from loci distinct from the sense transcript-encoding gene loci (protein-coding or non-protein-coding) are named long intergenic non-coding RNAs (lincRNAs). Exceptionally long lncRNAs are named macroRNAs and very long intergenic non-coding RNAs (vlincRNAs). Bidirectional transcription from the enhancer and promoter regions generates enhancer-associated RNAs (eRNAs) and promoter-associated long RNAs (PALRs), respectively. Antisense transcription can generate natural antisense transcripts (NATs) with varying degrees of overlap. NATs can overlap with sense transcripts at their 5′-ends (divergent NAT or head-to-head) or 3′-ends (convergent NAT or tail-to-tail). NATs and other lncRNAs can be expressed in linear or circular form. Modified with permission from Kung et al [4].

The advent of high-throughput genomic technologies such as microarrays and next-generation sequencing has facilitated the discovery of the complexity of the eukaryotic transcriptome [4-7]. Up to 90% of the human genome is transcribed, but only a small percentage of the transcribed genes encode proteins [8]; therefore, the pervasive transcription generates many non-protein-coding transcripts, referred by many as “dark matter RNAs” [9], whose functions remain largely unclear [10-13]. Several studies have provided an accurate landscape of the genomic context of lncRNAs [2, 14], but, as St Laurent and colleagues pointed out, these RNAs cannot be easily standardized due to “insufficient theoretical basis to classify and categorize the dark matter transcripts” [15]. Based on the current literature, we provide an updated classification of lncRNAs (Fig. 1). LncRNAs are generally defined as transcripts longer than 200 nucleotides that generally lack protein-coding potential and can be processed like mRNAs, i.e. spliced and polyadenylated [16]. According to the GENCODE v7 catalog, human lncRNAs can be divided into two main categories: the intergenic lncRNAs [4] and the genic or intragenic lncRNAs [2, 17]. The widespread transcription of long intergenic regions generates molecules named long intergenic non-coding RNAs (lincRNAs) or stand-alone lncRNAs which transcriptional unit, thus, do not overlap protein-coding genes [18, 19]. LincRNAs are usually spliced and polyadenylated, and they can range from hundreds of nucleotides to several kb in length [18, 19]. Many of the known lincRNAs are associated with the polycomb repressive complex 2 (PRC-2) or other chromatin-modifying complexes, suggesting that these transcripts are involved in transcriptional control by functioning as scaffolds for chromatin remodelling proteins [18, 19]. It is to note, however, that some lncRNAs are exceptionally long, like, for instance, *Airn* and *Kcnq1ot1* (108 and 91.5 kb in length, respectively) and some refers to these molecules as macroRNAs [15, 20]. While Koerner and colleagues define macroRNAs as “ncRNAs that can be as short as a few hundred nucleotides or as long as several hundred thousand nucleotides, the function of which does not depend on processing into short or micro RNAs” [21], essentially assimilating the term “macro” to the term “long”, St Laurent and colleagues seem to consider macroRNAs as transcripts that are tens of kb in length [15]. MacroRNAs are very long, mostly unspliced and nuclear transcripts that are transcribed by RNA polymerase II and, as in the case of the above mentioned *Airn* and *Kcnq1ot1*, are involved in the regulation of imprinting [20]. Very recently, it has been demonstrated that long stretches of the genome are transcribed to generate unexpectedly long transcripts named very long intergenic non-coding RNAs (vlincRNAs), some of which reach the astonishing length of 1Mb [15]. VlincRNAs are expressed in normal primary and embryonic stem cells (ESCs), and also in blood and tumor cells [15, 20]. VlincRNAs cover a wider portion of the genome than lincRNAs, but there is a low overlap between vlincRNAs and lincRNAs [15]: however, when vlincRNAs and lincRNAs overlap, it seems that the vlincRNA version is functional [15]. Although the role of vlincRNAs is not clear, they are supposed to be crucial for cellular balance as siRNA-mediated downregulation of selected vlincRNAs in K562 cell line provokes an increase in cell death [15]. Given the fact that vlincRNAs predominantly localize to the nucleus, it is possible that these long transcripts function as “intelligent scaffolds” to connect different portion of the genome [9, 15]. The scaffolding function of lincRNAs is well established, as in the case of *functional intergenic repeating RNA element* (*Firre)*, also known as *linc-RAP-1*: human *Firre* is transcribed from a 5 Mb gene locus located on the X chro­mosome and is expressed on both X chromosomes before and after X-chromosome inactivation [22]. *Firre* localizes in the nucleus and is characterized by a 156 bp repeating RNA domain (RRD) that serves for *Firre* interaction with heterogeneous nuclear ribonucleoprotein U (hnRNP-U). *Firre* and hnRNP-U coordinate the topological organization of multiple chromosomes, thus providing a trans-acting scaffold for multichromosomal interactions [22]. Scaffolding activity is one of the many that lincRNAs can exert: in fact, recent studies demonstrate a wide panel of functions for lincRNAs. As we will discuss more deeply in this paper, lincRNAs can function as “pseudotargets” for miRNAs (or competing endogenous RNA, ceRNA), thus avoiding their suppressive role on target RNAs [23-25]. LincRNAs can also compete for binding to proteins, thus preventing protein:protein interactions [26].

The pervasive transcription of the human genome occurs bidirectionally [12, 27], and this is particularly evident at promoter regions. RNA polymerase II promiscuously binds to gene enhancers, promoters, and transcription start and termination sites and generates several RNAs, including approximately 2 kb-long enhancer-associated RNAs (eRNAs), which are generally non-polyadenylated [28], and promoter-associated long RNAs (PALRs) [7, 11, 29]. While the role of PALRs remains unclear, there is now evidence that eRNAs promote chromatin opening and RNA polymerase II occupancy at and transcription of target genes [25, 30]. Conversely, the natural antisense transcripts (NATs) have been better studied and characterized. NATs are transcribed from the opposite strand of and are complementary to other RNA transcripts (sense) and usually undergo splicing and polyadenylation [31, 32]. Antisense transcription is enriched at both ends of sense genes [33, 34]. Therefore, NATs can be complementary to the 5′ or 3′ ends of sense mRNAs [35]: in the first case, the transcriptional machineries converge (head-to-head), and in the second, they diverge (tail-to-tail) [32]. Notably, the term “sense” used in the context of RNA often indicates protein-coding transcripts. Although NATs are mainly considered to be complementary to protein-coding RNAs, it is now well established that many lncRNAs possess an antisense counterpart, as in the case of the *X-inactive-specific transcript* (*Xist)*/*Tsix* transcript pair, which is involved in X-chromosome silencing (see below) [32, 36]. NATs can regulate gene expression by RNA:RNA interaction, thus modulating target RNA stability, splicing or translation [37-39], but also function as a scaffold for chromatin remodeling complexes such as PRC-2 [40-42].

Similarly to mRNAs, structural folding of lncRNAs is crucial for their functions [43]. Recently, it was discovered that many lncRNAs are expressed in a circular form [44, 45], indicating that lncRNAs, similar to proteins, can exert different roles depending on their isoforms, which highlights the magnitude of the functions performed by the “lncRNA world”.

### Inside the lncRNA world: a delicate balance of the factors involved in cell “stemness”, differentiation, adaptation and death

#### The X-factor: X chromosome inactivation as a paradigm for gene regulation by lncRNAs

The complexity of the lncRNA world is nowhere more evident than in the X-chromosome inactivation (XCI) process [46]. XCI in female mammals represses gene expression on one X-chromosome to maintain a balance between the sexes [47]. The X-inactivation center (XIC) is a region of the X-chromosome that encodes many lncRNAs, which drive its transcriptional silencing [48]. One of the first lncRNAs discovered and characterized in mammals is *Xist*, an approximately 17 kb molecule that is expressed only from the inactive X-chromosome [49]. *Xist* is essential for the initiation of XCI. *Xist* recruits PRC-2, a machinery that trimethylates histone H3 at Lys27 through a repeated motif named repeated A (RepA), thereby causing the epigenetic repression of the X-chromosome [46]. *Xist* activity is controlled by its antisense partner *Tsix*, which mediates the methylation of the *Xist* promoter by associating with DNA methyltransferase 3a (Dnmt3a) to silence *Xist* expression [41, 42]. An additional layer of complexity is the regulation of the *Xist*/*Tsix* sense-antisense pair by other non-coding transcripts, *X-inactivating intergenic transcript element* (*Xite*), *RepA* and *Just proximal to Xist (Jpx)*. *Xite* is a positive regulator of *Tsix* [50], whereas *RepA* and *Jpx* activate *Xist* to promote XCI [51, 52]. The work of Lee and colleagues and their review on non-coding “X-factors” underline the importance of lncRNAs, which adds a new perspective to the complexity of the non-coding RNA world [46].

#### Cell “stemness” and cell differentiation: two sides of the same coin controlled by lncRNAs

XCI is extremely important for cell differentiation and is regulated by many pluripotency factors, e.g., Oct-4, which activates *Tsix* and prevents XCI and cell differentiation [42, 53]. The differentiation of stem or progenitor cells depends on many factors that coordinate the expression of genes that drive the acquisition of lineage-specific features; the maintenance of stem cell pluripotency requires an equally specific molecular blueprint. In their milestone article, Takahashi and Yamanaka demonstrated that it is possible to reprogram differentiated cells to an embryonic-like state through the addition of few “core” transcription factors such as Oct-4 and Sox-2 [54]. Although most attention has focused on proteins, there is now growing evidence that lncRNAs play key roles in cell fate determination [55-57]. *LincRNA-regulator of reprogramming* (*linc-RoR*) is crucial for cellular reprogramming and the maintenance of pluripotency; it is highly expressed in induced pluripotent stem cells (iPS) and self-renewing human ESCs and is strongly downregulated during human ESC differentiation [58]. *Linc-RoR* positively regulates the expression of the core transcription factors Oct-4, Sox-2 and Nanog by functioning as a miRNA sponge (or ceRNA) [23]. Certain miRNAs such as *miR-145* directly target and repress the expression of pluripotency genes to inhibit human ESC self-renewal and induce lineage-restricted differentiation [59]; *linc-RoR* binds *miR-145*, thereby preventing the downregulation of Oct-4, Sox-2 and Nanog and human ESC differentiation [23].

Human genome consists of at least 50% repeat sequences, most of which derived from transposable elements [60]. Among them, human endogenous retroviruses (HERVs), which stem from ancient exogenous retroviral infection, account for 8% of the total human DNA [60]. More than 200 of the 1000 copies of *HERV-H* insertions in the human genomes are highly expressed in human ESCs, where they account for up to 2% of all polyadenylated transcripts [61]. *HERV-H* is an approximately 5 kb long lncRNA, which expression is essential for pluripotency maintenance of human ESCs; it has recently been demonstrated that knockdown of *HERV-H* in human ESCs results in upregulation of the pluripotency markers Oct4, Sox2 and Nanog, while the differentiation markers Gata6 and RunX1 are downregulated [62]. *HERV-H* functions as a scaffold to recruit Oct-4 and some transcriptional co-activators to the long terminal repeat (LTR) portion of its own gene loci, which in turn enhances the transcription of neighboring pluripotency-associated genes [62].

*Tcl1 upstream neuron-associated* (*TUNA*), an evolutionarily conserved transcript, contributes to ESCs maintenance and proliferation as well as neural commitment of ESCs [63]. *TUNA*, also known as *Megamind*, is located on chromosome 12 and is transcribed in the opposite direction to *Tcl1*: it is expressed in two isoforms of approximately 3 kb in length and it localizes in both nucleus and cytoplasm. ShRNA-mediated silencing of *TUNA* in mouse ESCs causes impaired cell proliferation, reduction of transcription of *Oct-4* and *Nanog*; conversely, its overexpression increases such pluripotency-related genes expression and associates with elevated levels of proliferation [63]. *TUNA* is highly expressed in the central nervous system of many vertebrates and plays a role in neural commitment: in fact, its levels are highly upregulated during neural differentiation of mouse ESCs and knockdown of *TUNA* renders mouse ESCs unable to differentiate. It is noteworthy that *TUNA* is expressed at high level in the thalamus and striatum in the human brain and may have a role in the pathophysiology of Huntington's Disease [63]. *TUNA* activates transcription of pluripotency-related genes by binding to their promoters and recruiting RNA Binding Proteins (RBPs) such as polypyrimidine tract-binding protein 1 (PTBP1) and heterogeneous nuclear ribonucleoprotein K (hnRNP-K) [63].

*Mistral*, also known as *Mira*, is an approximately 0.8 kb non-coding transcript that is generated from the genomic region between the *Hoxa6* and *Hoxa7* genes and drives the expression of the genes that are involved in germ-layer specification in differentiating mouse ESCs and are not expressed in pluripotent mouse ESCs [64]. *Mistral* is induced following treatment with retinoid acid, a potent inducer of differentiation, and recruits the epigenetic activator Mixed Lineage Leukemia 1 (MLL), thereby activating the expression of Hoxa6 and Hoxa7 [64]. This causes the upregulation of the genes expressed during early germ-layer differentiation; the siRNA-mediated knockdown of *Mistral* prevents this induction [64].

Many essential protein-coding genes also encode lncRNAs [65]. For instance, it is well established that lncRNAs are involved in the process of muscle differentiation. The *steroid receptor RNA activator* (*SRA*), an approximately 0.8 kb long transcript, was the first lncRNA whose secondary structure was solved [43, 66], and it was originally characterized as a non-coding RNA that contributed to the activation of several sex hormone receptors [67]. However, an *SRA* isoform was identified that encodes the steroid receptor RNA activator protein (SRAP) [68]; both *SRA* and SRAP play a role in cell differentiation. Whereas *SRA* co-activates the myogenic differentiation 1 (MyoD) protein to enhance myogenic differentiation and the myogenic conversion of non-muscle cells, SRAP prevents this co-activation by interacting with *SRA* [68]. Interestingly, a 24 kb regulatory region upstream of the *MyoD* gene locus encodes for several eRNAs that are essential for MyoD expression, in particular the *DNA enhancer elements Distal Regulatory Regions RNA* (*^DRR^RNA*) and *Core Enhancer RNA* (*^CE^RNA*) [69]. *^CE^RNA* contributes to chromatin remodeling at *MyoD*, thus promoting RNA polymerase II occupancy and transcription, while *^DRR^RNA* is an activator of the downstream myogenic genes [69]. Cesana and colleagues revealed the existence of a long intergenic transcript named *lincRNA Muscle Differentiation 1* (*linc-MD1*) that is involved in myoblast differentiation [25]. Human *linc-MD1* maps to chromosome 6p12.2; this transcript is polyadenylated and localizes in the cytoplasm, and its expression is induced upon myoblast differentiation [25]. Similar to *linc-RoR*, but with a pro-differentiation role, *linc-MD1* functions as a ceRNA by binding *miR-133* and *miR-135*, which target and inhibit the factors involved in myoblast differentiation [25]. Whereas *linc-RoR* prevents the miRNA-mediated repression of “stemness” factors, contributing to the maintenance of cell pluripotency and self-renewal, *linc-MD1* sequesters miRNAs that repress the expression of myogenic factors, thereby enhancing muscular differentiation [25]. It has recently been demonstrated that the RBP human antigen R (HuR), which expression is inhibited by *miR-133*, intersects the *linc-MD1*-miRNAs network creating a regulative loop with *linc-MD1* [70]. HuR and *linc-MD1* levels increase during early phases of myogenesis, while they decrease as differentiation program progresses. HuR binds *linc-MD1* promoting its sponging activity and *linc-MD1*, in turn, attenuates this effect by sequestering *miR-133* [70]. Recently, Kretz and colleagues identified two lncRNAs named *anti-differentiation ncRNA* (*ANCR*) and *tissue differentiation-inducing non-protein coding RNA* (*TINCR*) that are involved in epidermal differentiation [71, 72]. The *ANCR* gene maps to chromosome 4 and encodes an approximately 0.8 kb transcript whose expression is suppressed during keratinocyte, osteoblast and adipocyte differentiation [71, 73]. RNAi-mediated *ANCR* silencing in progenitor keratinocytes perturbs the expression of the genes associated with epidermal differentiation; for instance, *ANCR* depletion reduces the CEBPα level but increases the expression of the key epidermal differentiation proteins filaggrin, loricrin and keratin 1 [71]. In humans, the *TINCR* locus maps to chromosome 19 and generates a 3.7 kb transcript whose expression is strongly induced during epidermal differentiation [72]. The downregulation of *TINCR* causes the reduced expression of many genes during keratinocyte differentiation, including filaggrin and loricrin, and consequently causes a dramatic reduction in the ultra-structures that are essential for epidermal barrier formation, such as the protein-rich keratohyalin granules and lipid-rich lamellar bodies [72]. *TINCR* binds target mRNAs through a 25 nucleotide motif named the *TINCR* box [72]. Furthermore, *TINCR* strongly interacts with the staufen double-stranded RNA binding protein 1 (STAU-1), which mediates mRNA decay in cooperation with lncRNAs [74].

#### Fine tuning of cellular adaptation by lncRNAs

Cells are often exposed to stressors, like thermal stress and hypoxia, that necessitate the activation of molecular mechanisms of adaptation. The heat-shock response in vertebrates features several cytoprotective proteins including the heat-shock transcription factor 1 (HSF1), which is normally found as an inactive monomer in unstressed cells and trimerizes once activated by heat and other stress stimuli [75]. It was demonstrated that HSF1 trimerization is promoted by an approximately 0.6 kb long non-polyadenylated lncRNA named *heat shock RNA-1* (*HSR1*) [75]. *HSR1* interacts with the translation elongation factor eEF1A and promotes HSF1 trimerization and transcriptional activity. Inhibition of *HSR1* secondary structure or silencing of *HSR1* provokes a massive death in cell exposed to heat shock [75]. One of the critical aspects for cellular functions is oxygen availability which is fundamental for ATP generation; low oxygen tension causes a strong reduction in the ATP level and determines an overall repression of gene expression [76]. The main regulator of cellular response to low oxygen availability is the hypoxia inducible factor (HIF)-1, a heterodimer composed of two subunits, hypoxia-regulated HIF-1α and constitutively expressed HIF-1β: HIF-1 drives the transcription of the genes involved in the cellular adaptation to hypoxia, many of which are also important for cancer progression [77]. HIF-1α is regulated at the protein level and the RNA level by many trans-acting factors [78]. Trash-Bingham and Tartof discovered a natural antisense transcript named *antisense HIF* (*aHIF*) that is expressed in several adult and fetal tissues and in cancer tissues; *aHIF* is also upregulated in Von Hippel-Lindau (VHL)-negative kidney cancer [79, 80]. *aHIF* is approximately 2 kb long and does not have any apparent post-transcriptional modification; it is perfectly complementary to at least 860 bases of the 3′-end of the *HIF-1α* mRNA and contributes to the post-transcriptional regulation of this mRNA. Indeed, it was recently demonstrated that under hypoxia, *aHIF* negatively regulates *HIF-1α* mRNA by interfering with its translation [81, 82], although the exact mechanism of *aHIF* action remains unclear. It is known that hypoxia causes increased glycolysis and it was recently demonstrated that *lincRNA-p21* plays a role in this cell response [26]. *LincRNA-p21* is approximately 3 kb long: its transcriptional unit resides approximately 15 kb upstream of the *p21* gene locus and it is transcribed in the opposite orientation relative to *p21* [83]. At low oxygen tension HIF-1α up-regulates *lincRNA-p21* expression which in turn contributes to HIF-1α hypoxic accumulation by binding VHL and therefore preventing its binding to HIF-1α [26].

Hypoxia and other stimuli, such as DNA damage, starvation and oxidative stress, can activate the autophagic pathway as an adaptive response [84]. Autophagy is a vital process that degrades damaged cellular components and mediates their recycling; it plays an important role in somatic, stem and cancer cells and can also trigger apoptosis [85]. Although very little is known about the role of lncRNAs in autophagy, it was recently demonstrated that the knockdown of *maternally expressed gene 3* (*MEG3*) can activate autophagy and the proliferation of bladder cancer cells [86]. Human *MEG3* is an approximately 1.7 kb lncRNA that is expressed at high levels in brain tissues but at very low levels or is absent in cancer tissues and cell lines [87]. Interestingly, *MEG3* can also activate p53 via repression of the mouse double minute 2 homolog (MDM2), and the overexpression of *MEG3* in cancer cell lines suppresses cell proliferation [88].

#### Involvement of lncRNAs in apoptosis

Programmed cell death, also known as apoptosis, is a fundamental process that regulates tissue homeostasis. The tumor suppressor p53 and the transcription factors E2Fs play crucial roles in the response to several stimuli like, for instance, DNA damage, and are involved in cell cycle progression/arrest, autophagy and apoptosis [89-92]. Activation of E2F1 induces the expression of many lncRNAs including one named *E2F1-Regulated Inhibitor of Cell death* (*ERIC*), an approximately 1.7 kb long transcript [93]. *ERIC* is also upregulated by etoposide-induced DNA damage attenuating apoptotic response and may thus have cancer promoting effects [93]. The *WD repeat containing, antisense to p53* (*Wrap53*) gene encodes a protein that is essential for the formation of Cajal bodies, which are nuclear structures involved in ribonucleoprotein and small-nucleolar RNA (snRNA) processing [94]. The Wrap53 protein interacts with many small Cajal body-specific RNAs (scaRNAs), particularly with the *telomerase RNA component* (*TERC*), and telomerase reverse transcriptase (TERT), because it promotes telomerase localization in Cajal bodies [95]. Interestingly, the *Wrap53* gene also encodes for an antisense transcript that exists in three alternatively spliced forms, *Wrap53α*, *β* and *γ*: only *Wrap53α*, though, is complementary to the first exon of p53. *Wrap53α* positively regulates p53 expression by targeting and stabilizing the 5′ untranslated region of its mRNA: in fact, whereas *Wrap53α* silencing causes the downregulation of p53 mRNA and protein expression, its overexpression potentiates p53-mediated apoptosis [37].

Recently, it was found that p53 induces the expression of several lncRNAs upon doxorubicin-induced DNA damage; among these, the aforementioned *lincRNA-p21* functions as a gene repressor in the p53 pathway [83]. *LincRNA-p21* mediates transcriptional repression via the physical interaction of a 780 nt region at its 5′-end with hnRNP-K; this complex binds to the promoters of target genes, thereby mediating their transcriptional repression and activating an apoptotic response to DNA damage [83]. Notably, *lincRNA-p21* can also inhibit the translation of the *CTTNB1* and *JUNB* mRNAs, and the previously mentioned HuR enhances *lincRNA-p21* degradation through the recruitment of Argonaute 2, a component of the RNA-induced silencing complex (RISC) that cleaves target mRNAs [96]. DNA damage also stimulates the expression of several lncRNAs in a p53-independent manner: for instance, *JADE1 adjacent regulatory RNA (lncRNA-JADE)* is transcriptionally activated in MCF7 breast cancer cells following DNA damage induction in ATM/NFkB-dependent fashion [97]. The *lncRNA-JADE* gene is adjacent to *JADE1* gene and generates an approximately 1.7 kb long transcript which is highly conserved across mammalian species. JADE1 is a component of a multi-protein complex that drives H4 histone acetylation: *lncRNA-JADE* recruits p300 and BRCA1 to *JADE1* promoter and activates its transcription. Silencing of *lncRNA-JADE* impairs cell proliferation and increases apoptosis, while its overexpression enhances cell growth and diminishes apoptosis [97]. Notably, breast cancer tissues display a high level of *lncRNA-JADE* compared to normal breast tissue, suggesting that this transcript may play an important role in breast carcinogenesis and resistance to DNA-damaging chemotherapeutic drugs [97].

As previously mentioned, low oxygen tension is a stress cells often have to deal with and it was very recently showed that anoxia causes mitochondrial fission and apoptosis in cardiomiocytes [98]. Mitochondrial fission and apoptosis are inhibited by prohibitin 2 (PHB2), but anoxia also upregulates the expression of certain miRNAs, including *miR-539* which provokes downregulation of PHB2 [98]. Anoxia also downregulates many lncRNAs, including one named *cardiac apoptosis-related lncRNA* (*CARL*) which is involved in PHB2 control [98]. *CARL* functions as a sponge for *miR-539*: in fact, *CARL* overexpression reduces miR-539 expression and activity, increases PHD2 levels and attenuates anoxia-induced mitochondrial fission and apoptosis in cardiomiocytes [98].

### Dysregulated expression of untranslated RNAs: involvement of lncRNAs in the onset of pathologies and in the hallmark capabilities of cancer cells

LncRNAs are crucial for maintaining cellular physiology, and disequilibrium in the balance of lncRNAs could significantly contribute to the onset and development of several pathologies. Table 1 shows selected lncRNAs that are involved in human pathologies. Many reviews have highlighted the potential roles of lncRNAs in human diseases [99, 100], and lncRNAs are mainly associated with three classes of pathologies: neurodegenerative disorders [17], such as Huntington's [101] and Alzheimer's [102] diseases, cardiovascular diseases [103] and cancer [104, 105]. For example, it is well known that the production of peptides derived from the cleavage of the amyloid precursor protein by β-secretase-1 (BACE1) is a leading cause of Alzheimer's disease [106]. An antisense transcript of *BACE1* that positively regulates BACE1 was recently characterized [106, 107]. As for cardiovascular diseases, the massive dysregulation of lncRNAs has been observed in ventricular septal defects, which are the most common form of congenital heart disease [108], and the long polyadenylated *antisense noncoding RNA in the INK4 locus* (*ANRIL*) is strongly associated with cardiovascular diseases and other pathologies [109].

**Table 1:** Selected lncRNAs involved in human pathologies

LncRNA	Classification	Affected genes	Diseases	References
***AFAP1-AS1***	NAT	*AFAP1*	Cancer	[158]
***aHIF***	Convergent NAT	*HIF1a*	Cancer	[80, 153]
***ANRIL***	NAT	*INK4A, INK4B*	Atherosclerosis, cancer	[109, 119]
***ApoE AS1***	NAT	*ApoE*	Alzheimer's disease	[159]
***ATXN8OS***	Divergent NAT	*KLHL1*	Spinocerebellar ataxia type 8	[160]
***BACE1-AS***	NAT	*BACE1*	Alzheimer's disease	[106]
***BC200***	LincRNA	Many	Alzheimer's disease, Parkinson's disease, cancer	[161-163]
***Bcl2/IgH AS***	NAT	*Bcl2*	Cancer	[38]
***CRNDE***	LincRNA	Many	Cancer	[164, 165]
***DBE-T***	LincRNA	4q35 locus genes	Facioscapulohumeral muscular dystrophy	[166]
***DISC2***	NAT	*DISC1*	Schizophrenia	[167]
***FMR1 AS***	Linear NAT	*FMR1*	Fragile X syndrome	[168]
***FMR4***	Divergent PALR	*FMR1*	Fragile X syndrome	[169]
***FMR5***	Convergent PALR	*FMR1*	Fragile X syndrome	[170]
***FMR6***	Convergent 3′ NAT	*FMR1*	Fragile X syndrome	[170]
***GDNFOS1***	NAT	*GDNF*	Alzheimer's disease	[171]
***GDNFOS2***	NAT	*GDNF*	Alzheimer's disease	[171]
***Gomafu***	LincRNA	Many	Schizophrenia	[172]
***H19***	LincRNA	Many	Cancer	[173-175]
***HAR1F***	NAT	*HAR1R*	Huntington's disease	[176]
***HELLPAR***	LincRNA	Many	HELLP syndrome	[177]
***HOTAIR***	LincRNA	Many	Cancer	[149, 150]
***HTTAS***	NAT	*HTT*	Huntington's disease	[178]
***HULC***	LincRNA	Many	Cancer	[114, 152]
***LincRNA-p21***	LincRNA	Many	Cancer	[83]
***MALAT1***	LincRNA	Many	Cancer, diabetic retinopathy	[145, 174, 179]
***MVIH***	LincRNA	*PGK1*	Cancer	[135, 136]
***naPINK1***	NAT	*svPINK1*	Parkinson's disease, diabetes	[161, 180]
***NAT-RAD18***	NAT	*RAD18*	Alzheimer's disease	[181]
***NEAT1***	LincRNA	Many	Huntington's disease	[101]
***P15AS***	NAT	*P15*	Cancer	[182]
***PCAT-1***	LincRNA	Many	Cancer	[183]
***PRINS***	LincRNA	*G1P3*	Psoriasis	[184]
***PRNCR1***	LincRNA	Many	Cancer	[185, 186]
***Sox2OT***	Overlapping lncRNA	*Sox2*	Cancer, Alzheimer's disease, Parkinson's disease	[161, 187, 188]
***THRIL***	LincRNA	Many	Kawasaki disease	[189]
***TUG1***	LincRNA	Many	Huntington's disease, cancer	[161]
***UCA1***	LincRNA	Many	Cancer	[190, 191]

**Figure 2:**
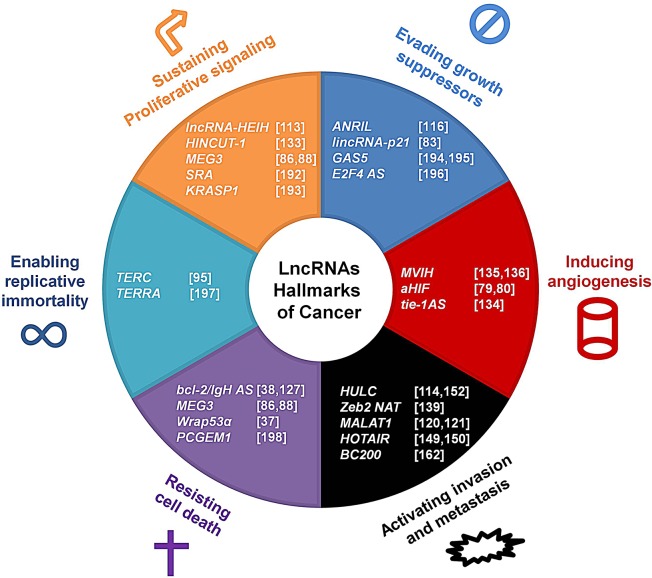
LncRNAs impact the hallmarks of cancer The six hallmarks of cancer are shown with selected associated lncRNAs that are involved in cancer onset and progression. References are listed in brackets.

The impact of dysregulated lncRNA expression is most evident in cancer, which is likely the most heterogeneous and unpredictable pathology. The pathogenesis of cancer is a multistep process due to the genetic alterations that perturb cellular physiology. The accumulation of mutations causes the evolution of tumor malignancy: as normal cells evolve to a neoplastic state, they acquire a succession of “hallmark capabilities” that enable the progression of the cancer [110]. There is an unexpected involvement of lncRNAs in several cellular processes; alterations in the expression of certain transcripts can lead to dramatic changes in the cellular physiology, leading to further consequences. There is growing evidence that lncRNAs play a role in cancer onset and development [105, 111]; indeed, lncRNAs are involved in the canonical hallmarks of cancer that were first proposed in 2001 (Fig. 2) [112]. Recently, it was shown that the expression profiles of lncRNAs in cancer cells are significantly different from those in normal cells. For example, *lncRNA highly expressed in hepatocellular carcinoma* (*lncRNA-HEIH*) expression is higher in liver cancer and in cirrhotic liver samples compared to normal liver tissues [113]. LncRNA *highly upregulated in liver cancer* (*HULC*) is strongly upregulated in liver tumor samples and slightly upregulated in cirrhotic liver tissue and focal nodular hyperplasia compared to normal tissue [114], suggesting that the expression of a specific lncRNA may reflect or cause the grade of the alteration. Here, we discuss how lncRNAs and their dysregulated expression are associated with cancer onset and development.

#### Dysregulation of cell growth: reinterpreting the concept of oncogenes and tumor suppressor genes from a non-coding perspective

In their milestone review “The Hallmarks of Cancer,” Hanahan and Weimberg discuss that the most distinctive characteristic of tumor cells is most likely “their ability to sustain chronic proliferation” [110]. The *INK4A-ARF-INK4B* gene cluster (*INK4* locus) is located on human chromosome 9p21 and encodes three tumor suppressors genes that are also known as *p16INK4A*, *p14ARF* and *p15INK4B* [115]. Whereas p15 and p16 suppress cell growth by inhibiting cyclin-dependent kinase (CDK) 4 and 6, p14 inhibits MDM2, thereby activating p53. The *INK4* locus encodes *ANRIL,* which is transcribed from the antisense strand of *p15INK4B* [115]; *ANRIL* is approximately 3.8-kb long and is expressed in linear and circular isoforms [116]; furthermore, four major *ANRIL* isoform groups with four distinct transcriptions have recently been identified [117]. *ANRIL* directly interacts with PRC-2 component suppressor of zeste 12 (SUZ12), thereby selectively repressing *in cis p15INK4B* transcription [40]: accordingly, the silencing of *ANRIL* causes an increase in the p15INK4B level and a strong reduction in cell viability. However, *ANRIL* isoforms can also regulate gene expression *in trans*: in fact, its overexpression causes broad changes in the expression of genes distributed across the genome and promotes cell growth and metabolic activity [117]. Alterations in the *ANRIL* structure or expression contribute to the onset of a variety of pathologies, including cancer [109, 117-119].

The *Metastasis associated lung adenocarcinoma transcript 1 (MALAT-1)*, also known as *non-coding nuclear-enriched abundant transcript 2* (*NEAT2*), is an 8,7 kb transcript that is expressed at high levels in the normal pancreas and lungs and at varying levels in the prostate, colon and other organs but is absent in the skin, stomach, bone marrow and uterus [120]. *MALAT-1* has been primarily studied for its role in cancer cells migration, invasion and metastasis [120, 121], but, in addition, it plays an important role in cell cycle progression. Tripathi and colleagues reported that *MALAT-1* expression is low during G1 and G2 cell cycle phases and high during G1/S and mitosis in human normal and cancer cell lines; moreover, silencing of *MALAT-1* induces cellular senescence in human lung fibroblasts and provokes G0/G1 or G2/M phase arrest, depending on cell type [122]. *MALAT-1* localizes at nuclear speckles and modulates the alternative splicing of RNAs by interacting with several pre-mRNA splicing factors and controlling their level of phosphorylation [123]. It has been demonstrated that *MALAT-1*-depleted cells show mitotic arrest due to an impaired level of B-MYB, a transcription factor involved in mitotic progression. B-MYB is overexpressed in many cancers and its expression and/or RNA processing is controlled by *MALAT-1* [122].

The fusion protein Bcr-Abl derives from the rearrangement of chromosome 9 with chromosome 22 and is a feature of more than 90% of Chronic Myeloid Leukemia cases. Bcr-Abl is a pro-proliferative and antiapoptotic protein and its aberrant expression leads to the upregulation of many lncRNAs [124]. Silencing of *Bcr-Abl* provokes a downregulation of these lncRNAs; among them, *beta globin locus transcript 3 (non-protein coding)* (*lncRNA-BGL3*) acts as a tumor suppressor transcript acting as a ceRNA for those miRNAs that target the oncosuppressor PTEN, such as *miR-17*, *miR-20* and *miR-106*. Indeed, tumor growth is much slower in nude mice injected with *lncRNA-BGL3*-overexpressing K562 cell line than in control mice. Furthermore, bone marrow cells derived from transgenic mice overexpressing *lncRNA-BGL3* and infected with a retrovirus encoding for Bcr-Abl show a decreased transformation capacity compared to Bcr-Abl-expressing bone marrow cells from control mice [124].

These findings remark the role of lncRNAs in regulating cell growth and cell cycle progression and how dysregulations in their expression can lead to impaired cell proliferation: therefore, from a non-coding perspective, oncogenes and oncosuppressor genes could be called *lnc*-ogenes and *lnc*-osuppressor genes.

#### Evading apoptosis: how lncRNAs influence the cell death threshold

Alterations in cell death pathways are an important step in cancer progression and there is now evidence that lncRNAs play a role in this process [125]. A diminished sensitivity to apoptosis is a common feature of various cancers, such as B-cell lymphoma, which is characterized by the overexpression of Bcl-2 [126]. We discovered a *Bcl-2/IgH* antisense transcript (*Bcl2/IgH AS*) that is expressed in the t(14;18) but not in the t(14;18)-negative lymphoid cell lines [38]. This antisense transcript originates in the *IgH* locus, encompasses the t(14;18) fusion site and spans at least the complete 3′ UTR region of the *Bcl-2* mRNA [38]. The downregulation of the *Bcl-2/IgH AS* lowered *Bcl-2* gene expression and inhibited neoplastic cell growth by inducing apoptosis in the t(14;18) lymphoid cell lines, suggesting that this transcript might positively regulate *Bcl-2* expression [127]. We also demonstrated that the chimeric *Bcl-2/IgH* transcript stabilizes *Bcl-2* mRNA at the post-transcriptional level by masking a destabilizing adenine + uracil-rich element (ARE) located in the 3′-UTR of the *Bcl-2* mRNA [128].

An important regulator of apoptosis in lymphoid cells is the Fas-FasL system, which represents a major player in the extrinsic cell death pathway and its expression is often dysregulated in Non-Hodgkin Lymphomas [129]. Fas is expressed as a transmembrane (mFas) or soluble (sFas) protein, depending on alternative splicing of its mRNA which is driven by RNA binding motif protein 5 (RBM5)-dependent exon skipping [130]. While mFas levels varies in lymphoma cells, sFas levels are high in the serum of patients with malignant hematological disorders, and tumor cells are therefore less sensitive to FasL-induced apoptosis [131]. The *Fas* gene encodes an antisense transcript named *Fas-Antisense1* (*Fas-AS1*) which is involved in *Fas* alternative splicing process [39]: *Fas-AS1* sequesters RBM5 inhibiting exon 6 skipping. *Fas-AS1* expression is frequently repressed in lymphoma cell lines and primary lymphomas compared with controls, and ectopic overexpression of *Fas-AS1* determines a reduction of sFAS isoform and an increase of mFAS, thereby stimulating FasL-induced apoptosis [39]. *Fas-AS1* promoter is hypermethylated in lymphomas by the methyl transferase EZH2, which is upregulated in these tumors. Interestingly, treatment of lymphoma cells with the methyl transferase inhibitor DZNeP results in a reduced methylation of *Fas-AS1* promoter, a great increase of *Fas-AS1* expression and consequently a reduction of sFAS isoform and an increase of mFAS [39].

#### Angiogenesis, epithelial-mesenchymal transition and metastasis: involvement of lncRNAs in the progression of cancer malignancy

The dysregulated and rapid proliferation of cancer cells creates a local hypoxic microenvironment that is a common feature of many tumors [132]. Low oxygen tension activates the HIF transcription factors, which modulate the expression of many lncRNAs named *hypoxia-induced noncoding ultraconserved transcripts* (*HINCUTs*) that are involved in cell proliferation under hypoxic conditions [133]. Furthermore, HIFs play an important role in angiogenesis, which is regulated, at least in part, by non-coding transcripts [134]. Indeed, it was shown that the lncRNA *MVIH* (*lncRNA associated with microvascular invasion in HCC*) promotes tumor angiogenesis [135]. *MVIH* expression is high in hepatocellular carcinomas compared to normal tissues and targets phosphoglycerate kinase 1 (PGK1), a glycolytic enzyme that can inhibit angiogenesis when secreted by cells [135]. *MVIH* prevent PGK1 secretion, thereby activating angiogenesis, tumor growth and metastasis [135]. Furthermore, *MVIH* is as an independent risk factor for the poor recurrence-free survival of HCC patients after hepatectomy [135] and it and it has also been recently identified as a regulator of proliferation and invasion of tumor cells and as a poor prognostic biomarker in non-small cell lung cancer (NSCLC) [136].

Hypoxia also increases the expression of *MALAT-1* in endothelial cells, modulating their angiogenic properties [137]. Silencing of *MALAT-1* reduces HUVEC cell line proliferation and cell cycle progression lowering the level of cell cycle regulatory genes, but increase *in vitro* migration and angiogenesis [137]. Furthermore, *MALAT-1* knockout mice present a delay in vessel extension and a reduction in vessel density in the retina [137]. An important event in cancer progression is the epithelial-mesenchymal transition (EMT), a complex cellular process that is mainly driven by two crucial factors, Snail1 and Twist [138], and is characterized by the loss of the epithelial phenotype and the acquisition of mesenchymal characteristics. The molecular features of the EMT include the downregulated expression of cell-cell adhesion molecules such as E-cadherin; Snail-1 promotes the transcriptional repression of *E-cadherin* directly and indirectly by upregulating the expression of the transcription factors Zeb1 and Zeb2 [139]. Snail-1 strongly induces Zeb2 expression via the *Zeb2* NAT. Snail-1 upregulates *Zeb2* NAT expression, which prevents the splicing of an IRES-containing 5′-UTR intron; this allows the ribosome machinery to bind to the *ZEB2* mRNA and promote its translation [139]. The overexpression of Twist in a human breast epithelial cell line results in the altered expression of many lncRNAs; therefore, several other lncRNAs may be involved in the EMT [140]. The downregulation or inactivation of E-cadherin contributes to the invasion of cancer cells and enhances their metastatic potential [110]. Snail-1, Twist and Zeb1 are induced by TGF-β, a cytokine that is able to activate EMT and tumor invasion [141, 142]. TGF-β also modulates the expression of a multitude of lncRNAs and particularly up-regulates the levels of a non-coding transcript named *lncRNA activated by TGF-*β (*lncRNA-ATB*) [142]. *LncRNA-ATB* plays a notable role in EMT: in fact, its overexpression induces EMT and promotes invasion of cancer cell lines and it is also noteworthy that *lncRNA-ATB* levels are higher in hepatocellular carcinoma specimens from patients than in correspondent normal hepatic tissues. *LncRNA-ATB* promotes and sustains EMT and tumor invasion via two mechanisms: on one hand, *lncRNA-ATB* up-regulates Zeb1 and Zeb2 by competitively binding the *miR-200* family, which targets Zeb1 and Zeb2, thus acting as a ceRNA [141, 143]. On the other hand, *lncRNA-ATB* increases the stability of *IL-11* mRNA thereby promoting IL-11 autocrine pathway and STAT-3 activation, thus promoting EMT and the invasion-metastasis processes in HCC [142].

LncRNAs also play a role in inhibiting EMT and tumor invasion, as for the case of *BRAF-activated non-coding RNA* (*BANCR*). *BANCR* is an approximately 0.7 kb transcript which expression is strongly downregulated in NCLSC tissues compared with normal tissues [144]. Low levels of *BANCR* are associated with poor survival while high levels of *BANCR* indicate a better prognosis of NSCLC patients. Overexpression of *BANCR* promotes apoptosis and upregulates the expression of E-cadherin while decreases the levels of N-cadherin and Snail-1 in A549 cells, thus inhibiting cell migration and invasion [144]. Furthermore, *BANCR* suppresses NSCLC cell metastasis *in vivo*: in fact, nude mice injected with *BANCR*-overexpressing lung adenocarcinoma cells display a reduction of the number of metastatic nodules, confirming the potential tumor-suppressing role of *BANCR* [144].

Another well-studied lncRNA, the aforementioned *MALAT-1*, activates the migration, invasion and metastatic development of non-small cell lung cancer cells [120, 121, 145]. It has recently been demonstrated that *MALAT-1* is induced by TGF-β in bladder cancer cells and its level is highly upregulated in bladder cancer specimens [146]. *MALAT-1* binds to SUZ12 and represses the expression of E-cadherin. TGF-β promotes the association of *MALAT-1* and SUZ12, thereby promoting bladder cancer cells invasion and metastasis both *in vitro* and *in vivo* [146]. It is noteworthy, however, that *MALAT-1* knockout in human lung and liver cancer cell lines does not affect cell proliferation compared to the corresponding wild-type cell lines. Furthermore, *MALAT-1* knockout mice do not show any detectable developmental or lethality phenotype when kept under normal stress-free conditions [147]: MALAT-1 might thus be dispensable for normal development but highly important for cancer onset and progression.

*Hox transcript antisense intergenic RNA* (*HOTAIR*) is an approximately 2 kb spliced and polyadenylated RNA that is generated from the *HOXC* locus. *HOTAIR* binds to the PRC-2 complex, which induces the transcriptional silencing of the *HOXD* locus by trimethylation of histone H3 at lysine-27 [148]. The high expression of *HOTAIR* observed in colorectal cancer samples is associated with bad prognosis, indicating its importance in the metastatic development of cancer [149, 150].

### Prospects and Predictions

Research over the last two decades has illuminated the complexity of the transcriptome and led to the detection of many lncRNAs. While it is still valid to assume that proteins are the effectors of cellular processes, new roles for RNA have begun to emerge; several lines of evidence indicate that lncRNAs play important roles in the control of gene expression and the maintenance of cellular functions. The balanced expression of these transcripts appears to be crucial for the maintenance of cellular homeostasis; consequently, the dysregulated expression of lncRNAs contributes to the onset and progression of many types of pathology. The dysregulation of lncRNA expression in many pathologies highlights their potential use as diagnostic and prognostic factors [151] and therapeutic targets [100]. *HULC* is detected in the plasma of hepatocellular carcinoma patients, suggesting that it could be a useful diagnostic marker for this cancer [152]. *MALAT-1* is a prognostic biomarker for the metastatic development of lung cancers [145], and *aHIF* is a marker for poor disease-free survival in breast cancer [153]. Targeting lncRNAs poses an intriguing challenge for therapy [100]. We demonstrated that the *Bcl-2/IgH AS* discovered in our laboratory can be efficiently downregulated with synthetic oligonucleotides [127]. This results in the reduction of Bcl-2 expression and an induction of apoptosis in t(14;18)-negative lymphoid cell lines [127]. The RNAi-mediated knockdown of the *BACE1 antisense RNA* (*BACE1-AS*) induces the reduction of the *BACE1* mRNA and protein levels *in vitro* and *in vivo*, suggesting that *BACE1*-*AS* could be a candidate drug target [106]. However, further studies are required on the structural and functional aspects of lncRNA biology to assess their potential as therapeutic targets [100].

**Figure 3:**
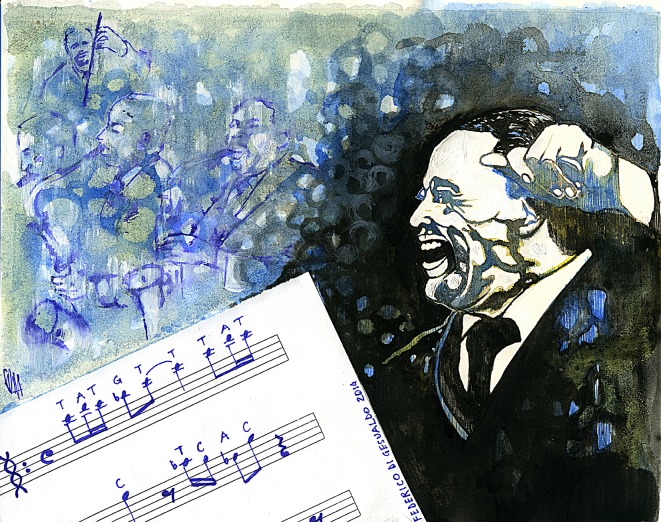
LncRNAs orchestrate the “diminuendo” and “crescendo” of cellular functions Duke Ellington was probably the greatest conductor, composer and arranger in jazz history. His longtime friend and colleague Billy Strayhorn once said: “Ellington plays piano. But his real instrument is his orchestra. Each member of his orchestra is to him a distinctive tone color and set of emotions, which he mixes with others equally distinctive to produce a third thing which I like to call the Ellington Effect” [199]. One of Ellington's most important compositions is “Diminuendo and Crescendo in Blue”: the title refers to expression marks that mean decreasing or increasing the sound in volume. The conductor controls the elements of musical expression (tempo, dynamics, articulation) as lncRNAs orchestrate and modulate gene expression and cellular functions. (Graphic: Federico Di Gesualdo).

The discovery of the fundamental roles of lncRNAs in the regulation of gene expression is both surprising and obvious, given that they can intrinsically interact with DNA using their nucleotide sequence and with proteins via secondary and tertiary structures [154, 155]. This cardinal role of lncRNAs evokes a “musical” analogy for the long non-coding RNA world (Fig. 3). An orchestra is an instrumental ensemble formed by many individual musicians. The complete score encodes all of the musical information; each section – or each player – possesses a timed blueprint, a part that can be read and translated into music using instruments. Each played part contributes to the fine and organized network of sounds. This parallels what occurs in cells as the DNA is transcribed to generate RNAs. mRNAs are translated via the ribosomal machinery to produce proteins, which act in a complex network of functions. However, a fine interpretation of the score needs someone who can coordinate the “diminuendo” and “crescendo” of each part: the conductor. Although the conductor does not produce sounds himself, his role is essential to coordinate all parts of the performance; similarly, lncRNAs are emerging as master regulators of cellular functions. Finally, the orchestral conductors are often exceptional player themselves, and in some cases, they perform with the other players. Recent studies have shown that lncRNAs are very rarely translated in cell lines [156] but are bound by ribosomes [157]. This finding raises questions about the effective coding potential/translation of lncRNAs and suggests further intricate scenarios; for example, lncRNAs might encode small active peptides or act as decoys for the translation machinery [157].

## List of abbreviations

ncRNA: non-coding RNA
lncRNA: long non-coding RNA
RNAi: RNA interference
lincRNA: long intergenic non-coding RNAs
vlincRNA: very long intergenic non-coding RNA
ESC: embryonic stem cell
eRNA: enhancer-associated RNA
PALR: promoter-associated long RNA
NAT: natural antisense transcript
Xist: X-inactive-specific transcript
XCI: X-chromosome inactivation
XIC: X-inactivation center
PRC-2: polycomb repressive complex 2
Firre: functional intergenic repeating RNA element
RRD: repeating RNA domain
hnRNP-U: heterogeneous nuclear ribonucleoprotein U
RepA: repeated A
Dnmt3a: DNA methyltransferase 3a
Xite: X-inactivating intergenic transcript element
JPX: just proximal to Xist
lincRoR: lincRNA-regulator of reprogramming
iPS: induced pluripotent stem cell
ceRNA: competing endogenous RNA
MLL: Mixed Lineage Leukemia 1
HERV: human endogenous retrovirus
LTR: long terminal repeat
TUNA: Tcl1 upstream neuron-associated
RBP: RNA Binding Protein
PTBP1: polypyrimidine tract-binding protein 1
hnRNP-K: heterogeneous nuclear ribonucleoprotein K
SRA: steroid receptor RNA activator
SRAP: steroid receptor RNA activator protein
Myo-D: myogenic differentiation 1
DRRRNA: DNA enhancer elements Distal Regulatory Regions RNA
CERNA: Core Enhancer RNA
linc-MD1: lincRNA Muscle Differentiation 1
HuR: human antigen R
ANCR: anti-differentiation ncRNA
TINCR: tissue differentiation-inducing non-protein coding RNA
STAU-1: staufen double-stranded RNA binding protein 1
HSF1: heat-shock transcription factor 1
HSR1: heat shock RNA-1
HIF: hypoxia inducible factor
VHL: Von Hippel–Lindau
aHIF: antisense HIF
MEG3: maternally expressed gene 3
MDM2: mouse double minute 2 homolog
ERIC: E2F1-Regulated Inhibitor of Cell death
Wrap53: WD repeat containing, antisense to p53
snRNA: small-nucleolar RNA
scaRNA: small Cajal body-specific RNA
TERC: telomerase RNA component
TERT: telomerase reverse transcriptase
RISC: RNA-induced silencing complex
lncRNA-JADE: JADE1 adjacent regulatory RNA
PHB2: prohibitin 2
CARL: cardiac apoptosis-related lncRNA
BACE1: β-secretase-1
ANRIL: antisense non-coding RNA in the INK4 locus
lncRNA-HEIH: lncRNA highly expressed in hepatocellular carcinoma
HULC: highly up-regulated in liver cancer
CDK: cyclin-dependent kinase
PRC-2: polycomb repressive complex 2
SUZ12: suppressor of zeste 12
MALAT-1: metastasis associated lung adenocarcinoma transcript 1
NEAT2: non-coding nuclear-enriched abundant transcript 2
lncRNA-BGL3: beta globin locus transcript 3 (non-protein coding)
Bcl-2/IgH AS: Bcl-2/IgH antisense transcript
ARE: adenine + uracil-rich element
RBM5: RNA binding motif protein 5 (RBM5)
Fas-AS1: Fas-Antisense1
HINCUTs: hypoxia-induced non-coding ultraconserved transcripts
MVIH: lncRNA associated with microvascular invasion in HCC
PGK1: phosphoglycerate kinase 1
NSCLC: non-small cell lung cancer
EMT: epithelial-mesenchymal transition
lncRNA-ATB: lncRNA activated by TGF-β
BANCR: BRAF-activated non-coding RNA
HOTAIR: Hox transcript antisense intergenic RNA
BACE1-AS: BACE1 antisense RNA

## References

[1] Amaral PP, Dinger ME, Mattick JS (2013). Non-coding RNAs in homeostasis, disease and stress responses: an evolutionary perspective. Brief Funct Genomics.

[2] Derrien T, Johnson R, Bussotti G, Tanzer A, Djebali S, Tilgner H, Guernec G, Martin D, Merkel A, Knowles DG, Lagarde J, Veeravalli L, Ruan X, Ruan Y, Lassmann T, Carninci P (2012). The GENCODE v7 catalog of human long noncoding RNAs: analysis of their gene structure, evolution, and expression. Genome Res.

[3] Sauvageau M, Goff LA, Lodato S, Bonev B, Groff AF, Gerhardinger C, Sanchez-Gomez DB, Hacisuleyman E, Li E, Spence M, Liapis SC, Mallard W, Morse M, Swerdel MR, D'Ecclessis MF, Moore JC (2013). Multiple knockout mouse models reveal lincRNAs are required for life and brain development. Elife.

[4] Kung JT, Colognori D, Lee JT (2013). Long noncoding RNAs: past, present, and future. Genetics.

[5] Carninci P, Kasukawa T, Katayama S, Gough J, Frith MC, Maeda N, Oyama R, Ravasi T, Lenhard B, Wells C, Kodzius R, Shimokawa K, Bajic VB, Brenner SE, Batalov S, Forrest AR (2005). The transcriptional landscape of the mammalian genome. Science.

[6] Mercer TR, Gerhardt DJ, Dinger ME, Crawford J, Trapnell C, Jeddeloh JA, Mattick JS, Rinn JL (2012). Targeted RNA sequencing reveals the deep complexity of the human transcriptome. Nat Biotechnol.

[7] Djebali S, Davis CA, Merkel A, Dobin A, Lassmann T, Mortazavi A, Tanzer A, Lagarde J, Lin W, Schlesinger F, Xue C, Marinov GK, Khatun J, Williams BA, Zaleski C, Rozowsky J (2012). Landscape of transcription in human cells. Nature.

[8] (2009). Post-transcriptional processing generates a diversity of 5′-modified long and short RNAs. Nature.

[9] Johnson JM, Edwards S, Shoemaker D, Schadt EE (2005). Dark matter in the genome: evidence of widespread transcription detected by microarray tiling experiments. Trends Genet.

[10] Cheng J, Kapranov P, Drenkow J, Dike S, Brubaker S, Patel S, Long J, Stern D, Tammana H, Helt G, Sementchenko V, Piccolboni A, Bekiranov S, Bailey DK, Ganesh M, Ghosh S (2005). Transcriptional maps of 10 human chromosomes at 5-nucleotide resolution. Science.

[11] Kapranov P, Cheng J, Dike S, Nix DA, Duttagupta R, Willingham AT, Stadler PF, Hertel J, Hackermuller J, Hofacker IL, Bell I, Cheung E, Drenkow J, Dumais E, Patel S, Helt G (2007). RNA maps reveal new RNA classes and a possible function for pervasive transcription. Science.

[12] Katayama S, Tomaru Y, Kasukawa T, Waki K, Nakanishi M, Nakamura M, Nishida H, Yap CC, Suzuki M, Kawai J, Suzuki H, Carninci P, Hayashizaki Y, Wells C, Frith M, Ravasi T (2005). Antisense transcription in the mammalian transcriptome. Science.

[13] Hangauer MJ, Vaughn IW, McManus MT (2013). Pervasive transcription of the human genome produces thousands of previously unidentified long intergenic noncoding RNAs. PLoS Genet.

[14] Costa FF (2010). Non-coding RNAs: Meet thy masters. Bioessays.

[15] St Laurent G, Shtokalo D, Dong B, Tackett MR, Fan X, Lazorthes S, Nicolas E, Sang N, Triche TJ, McCaffrey TA, Xiao W, Kapranov P (2013). VlincRNAs controlled by retroviral elements are a hallmark of pluripotency and cancer. Genome Biol.

[16] Guil S, Esteller M (2012). Cis-acting noncoding RNAs: friends and foes. Nat Struct Mol Biol.

[17] Salta E, De Strooper B (2012). Non-coding RNAs with essential roles in neurodegenerative disorders. Lancet Neurol.

[18] Guttman M, Amit I, Garber M, French C, Lin MF, Feldser D, Huarte M, Zuk O, Carey BW, Cassady JP, Cabili MN, Jaenisch R, Mikkelsen TS, Jacks T, Hacohen N, Bernstein BE (2009). Chromatin signature reveals over a thousand highly conserved large non-coding RNAs in mammals. Nature.

[19] Khalil AM, Guttman M, Huarte M, Garber M, Raj A, Rivea Morales D, Thomas K, Presser A, Bernstein BE, van Oudenaarden A, Regev A, Lander ES, Rinn JL (2009). Many human large intergenic noncoding RNAs associate with chromatin-modifying complexes and affect gene expression. Proc Natl Acad Sci U S A.

[20] Latos PA, Barlow DP (2009). Regulation of imprinted expression by macro non-coding RNAs. RNA Biol.

[21] Koerner MV, Pauler FM, Huang R, Barlow DP (2009). The function of non-coding RNAs in genomic imprinting. Development.

[22] Hacisuleyman E, Goff LA, Trapnell C, Williams A, Henao-Mejia J, Sun L, McClanahan P, Hendrickson DG, Sauvageau M, Kelley DR, Morse M, Engreitz J, Lander ES, Guttman M, Lodish HF, Flavell R (2014). Topological organization of multichromosomal regions by the long intergenic noncoding RNA Firre. Nat Struct Mol Biol.

[23] Wang Y, Xu Z, Jiang J, Xu C, Kang J, Xiao L, Wu M, Xiong J, Guo X, Liu H (2013). Endogenous miRNA sponge lincRNA-RoR regulates Oct4, Nanog, and Sox2 in human embryonic stem cell self-renewal. Dev Cell.

[24] Salmena L, Poliseno L, Tay Y, Kats L, Pandolfi PP (2011). A ceRNA hypothesis: the Rosetta Stone of a hidden RNA language?. Cell.

[25] Cesana M, Cacchiarelli D, Legnini I, Santini T, Sthandier O, Chinappi M, Tramontano A, Bozzoni I (2011). A long noncoding RNA controls muscle differentiation by functioning as a competing endogenous RNA. Cell.

[26] Yang F, Zhang H, Mei Y, Wu M (2014). Reciprocal regulation of HIF-1alpha and lincRNA-p21 modulates the Warburg effect. Mol Cell.

[27] Morris KV, Santoso S, Turner AM, Pastori C, Hawkins PG (2008). Bidirectional transcription directs both transcriptional gene activation and suppression in human cells. PLoS Genet.

[28] Orom UA, Shiekhattar R (2013). Long noncoding RNAs usher in a new era in the biology of enhancers. Cell.

[29] Bernstein BE, Birney E, Dunham I, Green ED, Gunter C, Snyder M (2012). An integrated encyclopedia of DNA elements in the human genome. Nature.

[30] Melo CA, Drost J, Wijchers PJ, van de Werken H, de Wit E, Oude Vrielink JA, Elkon R, Melo SA, Léveillé N, Kalluri R, de Laat W, Agami R (2013). eRNAs are required for p53-dependent enhancer activity and gene transcription. Mol Cell.

[31] Magistri M, Faghihi MA, St Laurent G, Wahlestedt C (2012). Regulation of chromatin structure by long noncoding RNAs: focus on natural antisense transcripts. Trends Genet.

[32] Werner A, Carlile M, Swan D (2009). What do natural antisense transcripts regulate?. RNA Biol.

[33] Finocchiaro G, Carro MS, Francois S, Parise P, DiNinni V, Muller H (2007). Localizing hotspots of antisense transcription. Nucleic Acids Res.

[34] He Y, Vogelstein B, Velculescu VE, Papadopoulos N, Kinzler KW (2008). The antisense transcriptomes of human cells. Science.

[35] Baranello L, Bertozzi D, Fogli MV, Pommier Y, Capranico G (2010). DNA topoisomerase I inhibition by camptothecin induces escape of RNA polymerase II from promoter-proximal pause site, antisense transcription and histone acetylation at the human HIF-1alpha gene locus. Nucleic Acids Res.

[36] Werner A, Swan D (2010). What are natural antisense transcripts good for?. Biochem Soc Trans.

[37] Mahmoudi S, Henriksson S, Corcoran M, Mendez-Vidal C, Wiman KG, Farnebo M (2009). Wrap53, a natural p53 antisense transcript required for p53 induction upon DNA damage. Mol Cell.

[38] Capaccioli S, Quattrone A, Schiavone N, Calastretti A, Copreni E, Bevilacqua A, Canti G, Gong L, Morelli S, Nicolin A (1996). A bcl-2/IgH antisense transcript deregulates bcl-2 gene expression in human follicular lymphoma t(14;18) cell lines. Oncogene.

[39] Sehgal L, Mathur R, Braun FK, Wise JF, Berkova Z, Neelapu S, Kwak LW, Samaniego F (2014). FAS-antisense 1 lncRNA and production of soluble versus membrane Fas in B-cell lymphoma. Leukemia.

[40] Kotake Y, Nakagawa T, Kitagawa K, Suzuki S, Liu N, Kitagawa M, Xiong Y (2011). Long non-coding RNA ANRIL is required for the PRC2 recruitment to and silencing of p15(INK4B) tumor suppressor gene. Oncogene.

[41] Senner CE, Brockdorff N (2009). Xist gene regulation at the onset of X inactivation. Curr Opin Genet Dev.

[42] Do JT, Han DW, Gentile L, Sobek-Klocke I, Stehling M, Scholer HR (2008). Enhanced reprogramming of Xist by induced upregulation of Tsix and Dnmt3a. Stem Cells.

[43] Novikova IV, Hennelly SP, Sanbonmatsu KY (2012). Structural architecture of the human long non-coding RNA, steroid receptor RNA activator. Nucleic Acids Res.

[44] Memczak S, Jens M, Elefsinioti A, Torti F, Krueger J, Rybak A, Maier L, Mackowiak SD, Gregersen LH, Munschauer M, Loewer A, Ziebold U, Landthaler M, Kocks C, le Noble F, Rajewsky N (2013). Circular RNAs are a large class of animal RNAs with regulatory potency. Nature.

[45] Hentze MW, Preiss T (2013). Circular RNAs: splicing's enigma variations. Embo j.

[46] Lee JT (2012). Epigenetic regulation by long noncoding RNAs. Science.

[47] Clemson CM, McNeil JA, Willard HF, Lawrence JB (1996). XIST RNA paints the inactive X chromosome at interphase: evidence for a novel RNA involved in nuclear/chromosome structure. J Cell Biol.

[48] Froberg JE, Yang L, Lee JT (2013). Guided by RNAs: X-inactivation as a model for lncRNA function. J Mol Biol.

[49] Brown CJ, Hendrich BD, Rupert JL, Lafreniere RG, Xing Y, Lawrence J, Willard HF (1992). The human XIST gene: analysis of a 17 kb inactive X-specific RNA that contains conserved repeats and is highly localized within the nucleus. Cell.

[50] Ogawa Y, Lee JT (2003). Xite, X-inactivation intergenic transcription elements that regulate the probability of choice. Mol Cell.

[51] Zhao J, Sun BK, Erwin JA, Song JJ, Lee JT (2008). Polycomb proteins targeted by a short repeat RNA to the mouse X chromosome. Science.

[52] Sun S, Del Rosario BC, Szanto A, Ogawa Y, Jeon Y, Lee JT (2013). Jpx RNA activates Xist by evicting CTCF. Cell.

[53] Navarro P, Oldfield A, Legoupi J, Festuccia N, Dubois A, Attia M, Schoorlemmer J, Rougeulle C, Chambers I, Avner P (2010). Molecular coupling of Tsix regulation and pluripotency. Nature.

[54] Takahashi K, Yamanaka S (2006). Induction of pluripotent stem cells from mouse embryonic and adult fibroblast cultures by defined factors. Cell.

[55] Fatica A, Bozzoni I (2014). Long non-coding RNAs: new players in cell differentiation and development. Nat Rev Genet.

[56] Ghosal S, Das S, Chakrabarti J (2013). Long noncoding RNAs: new players in the molecular mechanism for maintenance and differentiation of pluripotent stem cells. Stem Cells Dev.

[57] Hu W, Alvarez-Dominguez JR, Lodish HF (2012). Regulation of mammalian cell differentiation by long non-coding RNAs. EMBO Rep.

[58] Loewer S, Cabili MN, Guttman M, Loh YH, Thomas K, Park IH, Garber M, Curran M, Onder T, Agarwal S, Manos PD, Datta S, Lander ES, Schlaeger TM, Daley GQ, Rinn JL (2010). Large intergenic non-coding RNA-RoR modulates reprogramming of human induced pluripotent stem cells. Nat Genet.

[59] Xu N, Papagiannakopoulos T, Pan G, Thomson JA, Kosik KS (2009). MicroRNA-145 regulates OCT4, SOX2, and KLF4 and represses pluripotency in human embryonic stem cells. Cell.

[60] Lander ES, Linton LM, Birren B, Nusbaum C, Zody MC, Baldwin J, Devon K, Dewar K, Doyle M, FitzHugh W, Funke R, Gage D, Harris K, Heaford A, Howland J, Kann L (2001). Initial sequencing and analysis of the human genome. Nature.

[61] Santoni FA, Guerra J, Luban J (2012). HERV-H RNA is abundant in human embryonic stem cells and a precise marker for pluripotency. Retrovirology.

[62] Lu X, Sachs F, Ramsay L, Jacques PE, Goke J, Bourque G, Ng HH (2014). The retrovirus HERVH is a long noncoding RNA required for human embryonic stem cell identity. Nat Struct Mol Biol.

[63] Lin N, Chang KY, Li Z, Gates K, Rana ZA, Dang J, Zhang D, Han T, Yang CS, Cunningham TJ, Head SR, Duester G, Dong PD, Rana TM (2014). An evolutionarily conserved long noncoding RNA TUNA controls pluripotency and neural lineage commitment. Mol Cell.

[64] Bertani S, Sauer S, Bolotin E, Sauer F (2011). The noncoding RNA Mistral activates Hoxa6 and Hoxa7 expression and stem cell differentiation by recruiting MLL1 to chromatin. Mol Cell.

[65] Bovolenta M, Erriquez D, Valli E, Brioschi S, Scotton C, Neri M, Falzarano MS, Gherardi S, Fabris M, Rimessi P, Gualandi F, Perini G, Ferlini A (2012). The DMD locus harbours multiple long non-coding RNAs which orchestrate and control transcription of muscle dystrophin mRNA isoforms. PLoS One.

[66] Novikova IV, Hennelly SP, Tung CS, Sanbonmatsu KY (2013). Rise of the RNA machines: exploring the structure of long non-coding RNAs. J Mol Biol.

[67] Lanz RB, McKenna NJ, Onate SA, Albrecht U, Wong J, Tsai SY, Tsai MJ, O'Malley BW (1999). A steroid receptor coactivator, SRA, functions as an RNA and is present in an SRC-1 complex. Cell.

[68] Hube F, Velasco G, Rollin J, Furling D, Francastel C (2011). Steroid receptor RNA activator protein binds to and counteracts SRA RNA-mediated activation of MyoD and muscle differentiation. Nucleic Acids Res.

[69] Mousavi K, Zare H, Dell'orso S, Grontved L, Gutierrez-Cruz G, Derfoul A, Hager GL, Sartorelli V (2013). eRNAs promote transcription by establishing chromatin accessibility at defined genomic loci. Mol Cell.

[70] Legnini I, Morlando M, Mangiavacchi A, Fatica A, Bozzoni I (2014). A feedforward regulatory loop between HuR and the long noncoding RNA linc-MD1 controls early phases of myogenesis. Mol Cell.

[71] Kretz M, Webster DE, Flockhart RJ, Lee CS, Zehnder A, Lopez-Pajares V, Qu K, Zheng GX, Chow J, Kim GE, Rinn JL, Chang HY, Siprashvili Z, Khavari PA (2012). Suppression of progenitor differentiation requires the long noncoding RNA ANCR. Genes Dev.

[72] Kretz M, Siprashvili Z, Chu C, Webster DE, Zehnder A, Qu K, Lee CS, Flockhart RJ, Groff AF, Chow J, Johnston D, Kim GE, Spitale RC, Flynn RA, Zheng GX, Aiyer S (2013). Control of somatic tissue differentiation by the long non-coding RNA TINCR. Nature.

[73] Zhu L, Xu PC (2013). Downregulated LncRNA-ANCR promotes osteoblast differentiation by targeting EZH2 and regulating Runx2 expression. Biochem Biophys Res Commun.

[74] Gong C, Maquat LE (2011). lncRNAs transactivate STAU1-mediated mRNA decay by duplexing with 3′ UTRs via Alu elements. Nature.

[75] Shamovsky I, Ivannikov M, Kandel ES, Gershon D, Nudler E (2006). RNA-mediated response to heat shock in mammalian cells. Nature.

[76] Semenza GL (2012). Hypoxia-inducible factors in physiology and medicine. Cell.

[77] Semenza GL (2012). Hypoxia-inducible factors: mediators of cancer progression and targets for cancer therapy. Trends Pharmacol Sci.

[78] Galban S, Gorospe M (2009). Factors interacting with HIF-1alpha mRNA: novel therapeutic targets. Curr Pharm Des.

[79] Thrash-Bingham CA, Tartof KD (1999). aHIF: a natural antisense transcript overexpressed in human renal cancer and during hypoxia. J Natl Cancer Inst.

[80] Rossignol F, Vache C, Clottes E (2002). Natural antisense transcripts of hypoxia-inducible factor 1alpha are detected in different normal and tumour human tissues. Gene.

[81] Poitz DM, Augstein A, Hesse K, Christoph M, Ibrahim K, Braun-Dullaeus RC, Strasser RH, Schmeisser A (2014). Regulation of the HIF-system in human macrophages--differential regulation of HIF-alpha subunits under sustained hypoxia. Mol Immunol.

[82] Uchida T, Rossignol F, Matthay MA, Mounier R, Couette S, Clottes E, Clerici C (2004). Prolonged hypoxia differentially regulates hypoxia-inducible factor (HIF)-1alpha and HIF-2alpha expression in lung epithelial cells: implication of natural antisense HIF-1alpha. J Biol Chem.

[83] Huarte M, Guttman M, Feldser D, Garber M, Koziol MJ, Kenzelmann-Broz D, Khalil AM, Zuk O, Amit I, Rabani M, Attardi LD, Regev A, Lander ES, Jacks T, Rinn JL (2010). A large intergenic noncoding RNA induced by p53 mediates global gene repression in the p53 response. Cell.

[84] Pan H, Cai N, Li M, Liu GH, Izpisua Belmonte JC (2013). Autophagic control of cell ‘stemness’. EMBO Mol Med.

[85] Choi AM, Ryter SW, Levine B (2013). Autophagy in human health and disease. N Engl J Med.

[86] Ying L, Huang Y, Chen H, Wang Y, Xia L, Chen Y, Liu Y, Qiu F (2013). Downregulated MEG3 activates autophagy and increases cell proliferation in bladder cancer. Mol Biosyst.

[87] Zhang X, Zhou Y, Mehta KR, Danila DC, Scolavino S, Johnson SR, Klibanski A (2003). A pituitary-derived MEG3 isoform functions as a growth suppressor in tumor cells. J Clin Endocrinol Metab.

[88] Zhou Y, Zhong Y, Wang Y, Zhang X, Batista DL, Gejman R, Ansell PJ, Zhao J, Weng C, Klibanski A (2007). Activation of p53 by MEG3 non-coding RNA. J Biol Chem.

[89] Beckerman R, Prives C (2010). Transcriptional regulation by p53. Cold Spring Harb Perspect Biol.

[90] Polager S, Ginsberg D (2008). E2F - at the crossroads of life and death. Trends Cell Biol.

[91] Polager S, Ofir M, Ginsberg D (2008). E2F1 regulates autophagy and the transcription of autophagy genes. Oncogene.

[92] Iaquinta PJ, Lees JA (2007). Life and death decisions by the E2F transcription factors. Curr Opin Cell Biol.

[93] Feldstein O, Nizri T, Doniger T, Jacob J, Rechavi G, Ginsberg D (2013). The long non-coding RNA ERIC is regulated by E2F and modulates the cellular response to DNA damage. Mol Cancer.

[94] Mahmoudi S, Henriksson S, Weibrecht I, Smith S, Soderberg O, Stromblad S, Wiman KG, Farnebo M (2010). WRAP53 is essential for Cajal body formation and for targeting the survival of motor neuron complex to Cajal bodies. PLoS Biol.

[95] Venteicher AS, Abreu EB, Meng Z, McCann KE, Terns RM, Veenstra TD, Terns MP, Artandi SE (2009). A human telomerase holoenzyme protein required for Cajal body localization and telomere synthesis. Science.

[96] Yoon JH, Abdelmohsen K, Srikantan S, Yang X, Martindale JL, De S, Huarte M, Zhan M, Becker KG, Gorospe M (2012). LincRNA-p21 suppresses target mRNA translation. Mol Cell.

[97] Wan G, Hu X, Liu Y, Han C, Sood AK, Calin GA, Zhang X, Lu X (2013). A novel non-coding RNA lncRNA-JADE connects DNA damage signalling to histone H4 acetylation. Embo j.

[98] Wang K, Long B, Zhou LY, Liu F, Zhou QY, Liu CY, Fan YY, Li PF (2014). CARL lncRNA inhibits anoxia-induced mitochondrial fission and apoptosis in cardiomyocytes by impairing miR-539-dependent PHB2 downregulation. Nat Commun.

[99] Shi X, Sun M, Liu H, Yao Y, Song Y (2013). Long non-coding RNAs: a new frontier in the study of human diseases. Cancer Lett.

[100] Wahlestedt C (2013). Targeting long non-coding RNA to therapeutically upregulate gene expression. Nat Rev Drug Discov.

[101] Johnson R (2012). Long non-coding RNAs in Huntington's disease neurodegeneration. Neurobiol Dis.

[102] Tan L, Yu JT, Hu N (2013). Non-coding RNAs in Alzheimer's disease. Mol Neurobiol.

[103] Schonrock N, Harvey RP, Mattick JS (2012). Long noncoding RNAs in cardiac development and pathophysiology. Circ Res.

[104] Chen G, Wang Z, Wang D, Qiu C, Liu M, Chen X, Zhang Q, Yan G, Cui Q (2013). LncRNADisease: a database for long-non-coding RNA-associated diseases. Nucleic Acids Res.

[105] Deng G, Sui G (2013). Noncoding RNA in oncogenesis: a new era of identifying key players. Int J Mol Sci.

[106] Faghihi MA, Modarresi F, Khalil AM, Wood DE, Sahagan BG, Morgan TE, Finch CE, St Laurent G, Kenny PJ, Wahlestedt C (2008). Expression of a noncoding RNA is elevated in Alzheimer's disease and drives rapid feed-forward regulation of beta-secretase. Nat Med.

[107] Kang MJ, Abdelmohsen K, Hutchison ER, Mitchell SJ, Grammatikakis I, Guo R, Noh JH, Martindale JL, Yang X, Lee EK, Faghihi MA, Wahlestedt C, Troncoso JC, Pletnikova O, Perrone-Bizzozero N, Resnick SM (2014). HuD Regulates Coding and Noncoding RNA to Induce APP-->Abeta Processing. Cell Rep.

[108] Song G, Shen Y, Zhu J, Liu H, Liu M, Shen YQ, Zhu S, Kong X, Yu Z, Qian L (2013). Integrated analysis of dysregulated lncRNA expression in fetal cardiac tissues with ventricular septal defect. PLoS One.

[109] Bochenek G, Hasler R, El Mokhtari NE, Konig IR, Loos BG, Jepsen S, Rosenstiel P, Schreiber S, Schaefer AS (2013). The large non-coding RNA ANRIL, which is associated with atherosclerosis, periodontitis and several forms of cancer, regulates ADIPOR1, VAMP3 and C11ORF10. Hum Mol Genet.

[110] Hanahan D, Weinberg RA (2011). Hallmarks of cancer: the next generation. Cell.

[111] Morris KV, Vogt PK (2010). Long antisense non-coding RNAs and their role in transcription and oncogenesis. Cell Cycle.

[112] Gutschner T, Diederichs S (2012). The hallmarks of cancer: a long non-coding RNA point of view. RNA Biol.

[113] Yang F, Zhang L, Huo XS, Yuan JH, Xu D, Yuan SX, Zhu N, Zhou WP, Yang GS, Wang YZ, Shang JL, Gao CF, Zhang FR, Wang F, Sun SH (2011). Long noncoding RNA high expression in hepatocellular carcinoma facilitates tumor growth through enhancer of zeste homolog 2 in humans. Hepatology.

[114] Panzitt K, Tschernatsch MM, Guelly C, Moustafa T, Stradner M, Strohmaier HM, Buck CR, Denk H, Schroeder R, Trauner M, Zatloukal K (2007). Characterization of HULC, a novel gene with striking up-regulation in hepatocellular carcinoma, as noncoding RNA. Gastroenterology.

[115] Pasmant E, Laurendeau I, Heron D, Vidaud M, Vidaud D, Bieche I (2007). Characterization of a germ-line deletion, including the entire INK4/ARF locus, in a melanoma-neural system tumor family: identification of ANRIL, an antisense noncoding RNA whose expression coclusters with ARF. Cancer Res.

[116] Burd CE, Jeck WR, Liu Y, Sanoff HK, Wang Z, Sharpless NE (2010). Expression of linear and novel circular forms of an INK4/ARF-associated non-coding RNA correlates with atherosclerosis risk. PLoS Genet.

[117] Holdt LM, Hoffmann S, Sass K, Langenberger D, Scholz M, Krohn K, Finstermeier K, Stahringer A, Wilfert W, Beutner F, Gielen S, Schuler G, Gabel G, Bergert H, Bechmann I, Stadler PF (2013). Alu elements in ANRIL non-coding RNA at chromosome 9p21 modulate atherogenic cell functions through trans-regulation of gene networks. PLoS Genet.

[118] Bai Y, Nie S, Jiang G, Zhou Y, Zhou M, Zhao Y, Li S, Wang F, Lv Q, Huang Y, Yang Q, Li Q, Li Y, Xia Y, Liu Y, Liu J (2014). Regulation of CARD8 expression by ANRIL and association of CARD8 single nucleotide polymorphism rs2043211 (p. C10X) with ischemic stroke. Stroke.

[119] Zhang EB, Kong R, Yin DD, You LH, Sun M, Han L, Xu TP, Xia R, Yang JS, De W, Chen JF (2014). Long noncoding RNA ANRIL indicates a poor prognosis of gastric cancer and promotes tumor growth by epigenetically silencing of miR-99a/miR-449a. Oncotarget.

[120] Ji P, Diederichs S, Wang W, Boing S, Metzger R, Schneider PM, Tidow N, Brandt B, Buerger H, Bulk E, Thomas M, Berdel WE, Serve H, Muller-Tidow C (2003). MALAT-1, a novel noncoding RNA, and thymosin beta4 predict metastasis and survival in early-stage non-small cell lung cancer. Oncogene.

[121] Schmidt LH, Spieker T, Koschmieder S, Schaffers S, Humberg J, Jungen D, Bulk E, Hascher A, Wittmer D, Marra A, Hillejan L, Wiebe K, Berdel WE, Wiewrodt R, Muller-Tidow C (2011). The long noncoding MALAT-1 RNA indicates a poor prognosis in non-small cell lung cancer and induces migration and tumor growth. J Thorac Oncol.

[122] Tripathi V, Shen Z, Chakraborty A, Giri S, Freier SM, Wu X, Zhang Y, Gorospe M, Prasanth SG, Lal A, Prasanth KV (2013). Long noncoding RNA MALAT1 controls cell cycle progression by regulating the expression of oncogenic transcription factor B-MYB. PLoS Genet.

[123] Tripathi V, Ellis JD, Shen Z, Song DY, Pan Q, Watt AT, Freier SM, Bennett CF, Sharma A, Bubulya PA, Blencowe BJ, Prasanth SG, Prasanth KV (2010). The nuclear-retained noncoding RNA MALAT1 regulates alternative splicing by modulating SR splicing factor phosphorylation. Mol Cell.

[124] Guo G, Kang Q, Zhu X, Chen Q, Wang X, Chen Y, Ouyang J, Zhang L, Tan H, Chen R, Huang S, Chen JL (2014). A long noncoding RNA critically regulates Bcr-Abl-mediated cellular transformation by acting as a competitive endogenous RNA. Oncogene.

[125] Rossi MN, Antonangeli F (2014). LncRNAs: New Players in Apoptosis Control. Int J Cell Biol.

[126] Shortt J, Johnstone RW (2012). Oncogenes in cell survival and cell death. Cold Spring Harb Perspect Biol.

[127] Morelli S, Delia D, Capaccioli S, Quattrone A, Schiavone N, Bevilacqua A, Tomasini S, Nicolin A (1997). The antisense bcl-2-IgH transcript is an optimal target for synthetic oligonucleotides. Proc Natl Acad Sci U S A.

[128] Schiavone N, Rosini P, Quattrone A, Donnini M, Lapucci A, Citti L, Bevilacqua A, Nicolin A, Capaccioli S (2000). A conserved AU-rich element in the 3′ untranslated region of bcl-2 mRNA is endowed with a destabilizing function that is involved in bcl-2 down-regulation during apoptosis. Faseb j.

[129] Xerri L, Bouabdallah R, Devilard E, Hassoun J, Stoppa AM, Birg F (1998). Sensitivity to Fas-mediated apoptosis is null or weak in B-cell non-Hodgkin's lymphomas and is moderately increased by CD40 ligation. Br J Cancer.

[130] Bonnal S, Martinez C, Forch P, Bachi A, Wilm M, Valcarcel J (2008). RBM5/Luca-15/H37 regulates Fas alternative splice site pairing after exon definition. Mol Cell.

[131] Hara T, Tsurumi H, Takemura M, Goto H, Yamada T, Sawada M, Takahashi T, Moriwaki H (2000). Serum-soluble fas level determines clinical symptoms and outcome of patients with aggressive non-Hodgkin's lymphoma. Am J Hematol.

[132] Olivotto M, Dello Sbarba P (2008). Environmental restrictions within tumor ecosystems select for a convergent, hypoxia-resistant phenotype of cancer stem cells. Cell Cycle.

[133] Ferdin J, Nishida N, Wu X, Nicoloso MS, Shah MY, Devlin C, Ling H, Shimizu M, Kumar K, Cortez MA, Ferracin M, Bi Y, Yang D, Czerniak B, Zhang W, Schmittgen TD (2013). HINCUTs in cancer: hypoxia-induced noncoding ultraconserved transcripts. Cell Death Differ.

[134] Li K, Blum Y, Verma A, Liu Z, Pramanik K, Leigh NR, Chun CZ, Samant GV, Zhao B, Garnaas MK, Horswill MA, Stanhope SA, North PE, Miao RQ, Wilkinson GA, Affolter M (2010). A noncoding antisense RNA in tie-1 locus regulates tie-1 function *in vivo*. Blood.

[135] Yuan SX, Yang F, Yang Y, Tao QF, Zhang J, Huang G, Wang RY, Yang S, Huo XS, Zhang L, Wang F, Sun SH, Zhou WP (2012). Long noncoding RNA associated with microvascular invasion in hepatocellular carcinoma promotes angiogenesis and serves as a predictor for hepatocellular carcinoma patients' poor recurrence-free survival after hepatectomy. Hepatology.

[136] Nie FQ, Zhu Q, Xu TP, Zou YF, Xie M, Sun M, Xia R, Lu KH (2014). Long non-coding RNA MVIH indicates a poor prognosis for non-small cell lung cancer and promotes cell proliferation and invasion. Tumour Biol.

[137] Michalik KM, You X, Manavski Y, Doddaballapur A, Zornig M, Braun T, John D, Ponomareva Y, Chen W, Uchida S, Boon RA, Dimmeler S (2014). Long noncoding RNA MALAT1 regulates endothelial cell function and vessel growth. Circ Res.

[138] Wang Y, Shi J, Chai K, Ying X, Zhou BP (2013). The Role of Snail in EMT and Tumorigenesis. Curr Cancer Drug Targets.

[139] Beltran M, Puig I, Pena C, Garcia JM, Alvarez AB, Pena R, Bonilla F, de Herreros AG (2008). A natural antisense transcript regulates Zeb2/Sip1 gene expression during Snail1-induced epithelial-mesenchymal transition. Genes Dev.

[140] Hu P, Yang J, Hou Y, Zhang H, Zeng Z, Zhao L, Yu T, Tang X, Tu G, Cui X, Liu M (2014). LncRNA expression signatures of twist-induced epithelial-to-mesenchymal transition in MCF10A cells. Cell Signal.

[141] Gregory PA, Bracken CP, Smith E, Bert AG, Wright JA, Roslan S, Morris M, Wyatt L, Farshid G, Lim YY, Lindeman GJ, Shannon MF, Drew PA, Khew-Goodall Y, Goodall GJ (2011). An autocrine TGF-beta/ZEB/miR-200 signaling network regulates establishment and maintenance of epithelial-mesenchymal transition. Mol Biol Cell.

[142] Yuan JH, Yang F, Wang F, Ma JZ, Guo YJ, Tao QF, Liu F, Pan W, Wang TT, Zhou CC, Wang SB, Wang YZ, Yang Y, Yang N, Zhou WP, Yang GS (2014). A long noncoding RNA activated by TGF-beta promotes the invasion-metastasis cascade in hepatocellular carcinoma. Cancer Cell.

[143] Burk U, Schubert J, Wellner U, Schmalhofer O, Vincan E, Spaderna S, Brabletz T (2008). A reciprocal repression between ZEB1 and members of the miR-200 family promotes EMT and invasion in cancer cells. EMBO Rep.

[144] Sun M, Liu XH, Wang KM, Nie FQ, Kong R, Yang JS, Xia R, Xu TP, Jin FY, Liu ZJ, Chen JF, Zhang EB, De W, Wang ZX (2014). Downregulation of BRAF activated non-coding RNA is associated with poor prognosis for non-small cell lung cancer and promotes metastasis by affecting epithelial-mesenchymal transition. Mol Cancer.

[145] Gutschner T, Hammerle M, Eissmann M, Hsu J, Kim Y, Hung G, Revenko A, Arun G, Stentrup M, Gross M, Zornig M, MacLeod AR, Spector DL, Diederichs S (2013). The noncoding RNA MALAT1 is a critical regulator of the metastasis phenotype of lung cancer cells. Cancer Res.

[146] Fan Y, Shen B, Tan M, Mu X, Qin Y, Zhang F, Liu Y (2014). TGF-beta-induced upregulation of malat1 promotes bladder cancer metastasis by associating with suz12. Clin Cancer Res.

[147] Eissmann M, Gutschner T, Hammerle M, Gunther S, Caudron-Herger M, Gross M, Schirmacher P, Rippe K, Braun T, Zornig M, Diederichs S (2012). Loss of the abundant nuclear non-coding RNA MALAT1 is compatible with life and development. RNA Biol.

[148] Rinn JL, Kertesz M, Wang JK, Squazzo SL, Xu X, Brugmann SA, Goodnough LH, Helms JA, Farnham PJ, Segal E, Chang HY (2007). Functional demarcation of active and silent chromatin domains in human HOX loci by noncoding RNAs. Cell.

[149] Kogo R, Shimamura T, Mimori K, Kawahara K, Imoto S, Sudo T, Tanaka F, Shibata K, Suzuki A, Komune S, Miyano S, Mori M (2011). Long noncoding RNA HOTAIR regulates polycomb-dependent chromatin modification and is associated with poor prognosis in colorectal cancers. Cancer Res.

[150] Gupta RA, Shah N, Wang KC, Kim J, Horlings HM, Wong DJ, Tsai MC, Hung T, Argani P, Rinn JL, Wang Y, Brzoska P, Kong B, Li R, West RB, van de Vijver MJ (2010). Long non-coding RNA HOTAIR reprograms chromatin state to promote cancer metastasis. Nature.

[151] Sanchez Y, Huarte M (2013). Long non-coding RNAs: challenges for diagnosis and therapies. Nucleic Acid Ther.

[152] Xie H, Ma H, Zhou D (2013). Plasma HULC as a promising novel biomarker for the detection of hepatocellular carcinoma. Biomed Res Int.

[153] Cayre A, Rossignol F, Clottes E, Penault-Llorca F (2003). aHIF but not HIF-1alpha transcript is a poor prognostic marker in human breast cancer. Breast Cancer Res.

[154] St Laurent G, Wahlestedt C (2007). Noncoding RNAs: couplers of analog and digital information in nervous system function?. Trends Neurosci.

[155] Mattick JS (2007). A new paradigm for developmental biology. J Exp Biol.

[156] Banfai B, Jia H, Khatun J, Wood E, Risk B, Gundling WE, Kundaje A, Gunawardena HP, Yu Y, Xie L, Krajewski K, Strahl BD, Chen X, Bickel P, Giddings MC, Brown JB (2012). Long noncoding RNAs are rarely translated in two human cell lines. Genome Res.

[157] Guttman M, Russell P, Ingolia NT, Weissman JS, Lander ES (2013). Ribosome profiling provides evidence that large noncoding RNAs do not encode proteins. Cell.

[158] Wu W, Bhagat TD, Yang X, Song JH, Cheng Y, Agarwal R, Abraham JM, Ibrahim S, Bartenstein M, Hussain Z, Suzuki M, Yu Y, Chen W, Eng C, Greally J, Verma A (2013). Hypomethylation of noncoding DNA regions and overexpression of the long noncoding RNA, AFAP1-AS1, in Barrett's esophagus and esophageal adenocarcinoma. Gastroenterology.

[159] Seitz A, Gourevitch D, Zhang XM, Clark L, Chen P, Kragol M, Levenkova N, Rux J, Samulewicz S, Heber-Katz E (2005). Sense and antisense transcripts of the apolipoprotein E gene in normal and ApoE knockout mice, their expression after spinal cord injury and corresponding human transcripts. Hum Mol Genet.

[160] Moseley ML, Zu T, Ikeda Y, Gao W, Mosemiller AK, Daughters RS, Chen G, Weatherspoon MR, Clark HB, Ebner TJ, Day JW, Ranum LP (2006). Bidirectional expression of CUG and CAG expansion transcripts and intranuclear polyglutamine inclusions in spinocerebellar ataxia type 8. Nat Genet.

[161] Wu P, Zuo X, Deng H, Liu X, Liu L, Ji A (2013). Roles of long noncoding RNAs in brain development, functional diversification and neurodegenerative diseases. Brain Res Bull.

[162] Iacoangeli A, Lin Y, Morley EJ, Muslimov IA, Bianchi R, Reilly J, Weedon J, Diallo R, Bocker W, Tiedge H (2004). BC200 RNA in invasive and preinvasive breast cancer. Carcinogenesis.

[163] Mus E, Hof PR, Tiedge H (2007). Dendritic BC200 RNA in aging and in Alzheimer's disease. Proc Natl Acad Sci U S A.

[164] Graham LD, Pedersen SK, Brown GS, Ho T, Kassir Z, Moynihan AT, Vizgoft EK, Dunne R, Pimlott L, Young GP, Lapointe LC, Molloy PL (2011). Colorectal Neoplasia Differentially Expressed (CRNDE), a Novel Gene with Elevated Expression in Colorectal Adenomas and Adenocarcinomas. Genes Cancer.

[165] Furney SJ, Pedersen M, Gentien D, Dumont AG, Rapinat A, Desjardins L, Turajlic S, Piperno-Neumann S, de la Grange P, Roman-Roman S, Stern MH, Marais R (2013). SF3B1 mutations are associated with alternative splicing in uveal melanoma. Cancer Discov.

[166] Cabianca DS, Casa V, Bodega B, Xynos A, Ginelli E, Tanaka Y, Gabellini D (2012). A long ncRNA links copy number variation to a polycomb/trithorax epigenetic switch in FSHD muscular dystrophy. Cell.

[167] Millar JK, Wilson-Annan JC, Anderson S, Christie S, Taylor MS, Semple CA, Devon RS, St Clair DM, Muir WJ, Blackwood DH, Porteous DJ (2000). Disruption of two novel genes by a translocation co-segregating with schizophrenia. Hum Mol Genet.

[168] Ladd PD, Smith LE, Rabaia NA, Moore JM, Georges SA, Hansen RS, Hagerman RJ, Tassone F, Tapscott SJ, Filippova GN (2007). An antisense transcript spanning the CGG repeat region of FMR1 is upregulated in premutation carriers but silenced in full mutation individuals. Hum Mol Genet.

[169] Khalil AM, Faghihi MA, Modarresi F, Brothers SP, Wahlestedt C (2008). A novel RNA transcript with antiapoptotic function is silenced in fragile X syndrome. PLoS One.

[170] Pastori C, Peschansky VJ, Barbouth D, Mehta A, Silva JP, Wahlestedt C (2014). Comprehensive analysis of the transcriptional landscape of the human FMR1 gene reveals two new long noncoding RNAs differentially expressed in Fragile X syndrome and Fragile X-associated tremor/ataxia syndrome. Hum Genet.

[171] Airavaara M, Pletnikova O, Doyle ME, Zhang YE, Troncoso JC, Liu QR (2011). Identification of novel GDNF isoforms and cis-antisense GDNFOS gene and their regulation in human middle temporal gyrus of Alzheimer disease. J Biol Chem.

[172] Barry G, Briggs JA, Vanichkina DP, Poth EM, Beveridge NJ, Ratnu VS, Nayler SP, Nones K, Hu J, Bredy TW, Nakagawa S, Rigo F, Taft RJ, Cairns MJ, Blackshaw S, Wolvetang EJ (2014). The long non-coding RNA Gomafu is acutely regulated in response to neuronal activation and involved in schizophrenia-associated alternative splicing. Mol Psychiatry.

[173] Li H, Yu B, Li J, Su L, Yan M, Zhu Z, Liu B (2014). Overexpression of lncRNA H19 enhances carcinogenesis and metastasis of gastric cancer. Oncotarget.

[174] Liu SP, Yang JX, Cao DY, Shen K (2013). Identification of differentially expressed long non-coding RNAs in human ovarian cancer cells with different metastatic potentials. Cancer Biol Med.

[175] Luo M, Li Z, Wang W, Zeng Y, Liu Z, Qiu J (2013). Long non-coding RNA H19 increases bladder cancer metastasis by associating with EZH2 and inhibiting E-cadherin expression. Cancer Lett.

[176] Johnson R, Richter N, Jauch R, Gaughwin PM, Zuccato C, Cattaneo E, Stanton LW (2010). Human accelerated region 1 noncoding RNA is repressed by REST in Huntington's disease. Physiol Genomics.

[177] van Dijk M, Thulluru HK, Mulders J, Michel OJ, Poutsma A, Windhorst S, Kleiverda G, Sie D, Lachmeijer AM, Oudejans CB (2012). HELLP babies link a novel lincRNA to the trophoblast cell cycle. J Clin Invest.

[178] Chung DW, Rudnicki DD, Yu L, Margolis RL (2011). A natural antisense transcript at the Huntington's disease repeat locus regulates HTT expression. Hum Mol Genet.

[179] Yan B, Tao Z-F, Li X-M, Zhang H, Yao J, Jiang Q (2014). Aberrant Expression of Long Noncoding RNAs in Early Diabetic Retinopathy.

[180] Scheele C, Nielsen AR, Walden TB, Sewell DA, Fischer CP, Brogan RJ, Petrovic N, Larsson O, Tesch PA, Wennmalm K, Hutchinson DS, Cannon B, Wahlestedt C, Pedersen BK, Timmons JA (2007). Altered regulation of the PINK1 locus: a link between type 2 diabetes and neurodegeneration?. Faseb j.

[181] Parenti R, Paratore S, Torrisi A, Cavallaro S (2007). A natural antisense transcript against Rad18, specifically expressed in neurons and upregulated during beta-amyloid-induced apoptosis. Eur J Neurosci.

[182] Yu W, Gius D, Onyango P, Muldoon-Jacobs K, Karp J, Feinberg AP, Cui H (2008). Epigenetic silencing of tumour suppressor gene p15 by its antisense RNA. Nature.

[183] Prensner JR, Iyer MK, Balbin OA, Dhanasekaran SM, Cao Q, Brenner JC, Laxman B, Asangani IA, Grasso CS, Kominsky HD, Cao X, Jing X, Wang X, Siddiqui J, Wei JT, Robinson D (2011). Transcriptome sequencing across a prostate cancer cohort identifies PCAT-1, an unannotated lincRNA implicated in disease progression. Nat Biotechnol.

[184] Szegedi K, Sonkoly E, Nagy N, Nemeth IB, Bata-Csorgo Z, Kemeny L, Dobozy A, Szell M (2010). The anti-apoptotic protein G1P3 is overexpressed in psoriasis and regulated by the non-coding RNA, PRINS. Exp Dermatol.

[185] Li L, Sun R, Liang Y, Pan X, Li Z, Bai P, Zeng X, Zhang D, Zhang L, Gao L (2013). Association between polymorphisms in long non-coding RNA PRNCR1 in 8q24 and risk of colorectal cancer. J Exp Clin Cancer Res.

[186] Yang L, Lin C, Jin C, Yang JC, Tanasa B, Li W, Merkurjev D, Ohgi KA, Meng D, Zhang J, Evans CP, Rosenfeld MG (2013). lncRNA-dependent mechanisms of androgen-receptor-regulated gene activation programs. Nature.

[187] Shahryari A, Rafiee MR, Fouani Y, Oliae NA, Samaei NM, Shafiee M, Semnani S, Vasei M, Mowla SJ (2014). Two novel splice variants of SOX2OT, SOX2OT-S1, and SOX2OT-S2 are coupregulated with SOX2 and OCT4 in esophageal squamous cell carcinoma. Stem Cells.

[188] Arisi I, D'Onofrio M, Brandi R, Felsani A, Capsoni S, Drovandi G, Felici G, Weitschek E, Bertolazzi P, Cattaneo A (2011). Gene expression biomarkers in the brain of a mouse model for Alzheimer's disease: mining of microarray data by logic classification and feature selection. J Alzheimers Dis.

[189] Li Z, Chao TC, Chang KY, Lin N, Patil VS, Shimizu C, Head SR, Burns JC, Rana TM (2014). The long noncoding RNA THRIL regulates TNFalpha expression through its interaction with hnRNPL. Proc Natl Acad Sci U S A.

[190] Yang C, Li X, Wang Y, Zhao L, Chen W (2012). Long non-coding RNA UCA1 regulated cell cycle distribution via CREB through PI3-K dependent pathway in bladder carcinoma cells. Gene.

[191] Han Y, Yang YN, Yuan HH, Zhang TT, Sui H, Wei XL, Liu L, Huang P, Zhang WJ, Bai YX (2014). UCA1, a long non-coding RNA up-regulated in colorectal cancer influences cell proliferation, apoptosis and cell cycle distribution. Pathology.

[192] Leygue E, Dotzlaw H, Watson PH, Murphy LC (1999). Expression of the steroid receptor RNA activator in human breast tumors. Cancer Res.

[193] Poliseno L, Salmena L, Zhang J, Carver B, Haveman WJ, Pandolfi PP (2010). A coding-independent function of gene and pseudogene mRNAs regulates tumour biology. Nature.

[194] Mourtada-Maarabouni M, Pickard MR, Hedge VL, Farzaneh F, Williams GT (2009). GAS5, a non-protein-coding RNA, controls apoptosis and is downregulated in breast cancer. Oncogene.

[195] Pickard MR, Williams GT (2014). Regulation of apoptosis by long non-coding RNA GAS5 in breast cancer cells: implications for chemotherapy. Breast Cancer Res Treat.

[196] Yochum GS, Cleland R, McWeeney S, Goodman RH (2007). An antisense transcript induced by Wnt/beta-catenin signaling decreases E2F4. J Biol Chem.

[197] Yu TY, Kao YW, Lin JJ (2014). Telomeric transcripts stimulate telomere recombination to suppress senescence in cells lacking telomerase. Proc Natl Acad Sci U S A.

[198] Fu X, Ravindranath L, Tran N, Petrovics G, Srivastava S (2006). Regulation of apoptosis by a prostate-specific and prostate cancer-associated noncoding gene, PCGEM1. DNA Cell Biol.

[199] Shapiro N, Hentoff N (1966). Hear Me Talkin’ to Ya: The Story of Jazz As Told by the Men Who Made It.

